# Construction and validation of immune prognosis model for lung adenocarcinoma based on machine learning

**DOI:** 10.3389/fonc.2025.1630663

**Published:** 2025-07-22

**Authors:** Jinyu Zheng, Xiaoyi Xu, Xianguo Chen, Xianshuai Li, Miao Fu, Yiping Zheng, Jie Yang

**Affiliations:** ^1^ Department of Cardiothoracic Surgery, Affiliated Jinhua Hospital, Zhejiang University School of Medicine, Jinhua, China; ^2^ Department of Clinical Laboratory, Affiliated Jinhua Hospital, Zhejiang University School of Medicine, Jinhua, China

**Keywords:** lung adenocarcinoma, immune-related markers, weighted gene co-expression network analysis, machine learning, prognostic model

## Abstract

**Introduction:**

Lung adenocarcinoma is a leading subtype of lung cancer with high rates of recurrence and metastasis. Identifying novel prognostic biomarkers is essential for improving patient outcomes.

**Methods:**

Transcriptomic and clinicopathological data from TCGA (55 tumor samples and 38 normal samples) were used to construct a prognostic model, with 30 samples for internal validation. An external validation cohort (10 tumor-normal pairs) was obtained from the First Affiliated Hospital of Wenzhou Medical University. Differentially expressed genes and immune-related genes from the IMMPORT database were integrated using WGCNA. Three machine learning algorithms—Random Forest, LASSO, and SVM-RFE—were applied to identify key hub genes. A multivariate Cox regression model was built to predict survival. Model performance was assessed by time-dependent ROC and ANN models. Immune infiltration was analyzed using TIMER and ssGSEA, with consensus clustering performed to explore immune subtypes. Protein expression and biological functions of hub genes were validated using the HPA database and GSEA.

**Results:**

A total of 1,822 DEGs were identified, with 68 immune-related genes significantly associated with LUAD prognosis. Four hub genes—CBLC, GDF10, LTBP4, and FABP4—were selected to construct the prognostic model, which showed strong predictive performance in both ROC and ANN analyses. Immune profiling revealed elevated CD4⁺ T cells, macrophages, and dendritic cells in LUAD. Consensus clustering identified two immune subtypes with distinct prognoses and immune landscapes.

**Discussion:**

This study established a robust immune-related prognostic model for LUAD and identified key biomarkers associated with immune infiltration and survival. These findings offer valuable insights for personalized diagnosis and treatment strategies in LUAD.

## 1 Introduction

Lung cancer remains the leading cause of cancer deaths (1, 2), with lung adenocarcinoma (LUAD) accounting for about 40% of cases and a poor five-year survival rate of 12%-15% (3). Early diagnosis is critical, but current methods often fail to predict recurrence or metastasis effectively. Therefore, identifying reliable prognostic biomarkers is urgently needed (4).

Immune-related genes (IRGs) have shown strong prognostic value in several cancers, but their role in LUAD remains underexplored, limiting immunotherapy advances. Discovering new IRGs could refine LUAD treatment and enhance personalized care.

Weighted Gene Co-expression Network Analysis (WGCNA) is a powerful tool that identifies gene modules related to clinical traits, uncovering disease mechanisms and novel biomarkers (5–8). Widely applied across fields, it enhances understanding beyond single-gene analyses.

Meanwhile, machine learning, including algorithms like Random Forest (RF), Least absolute shrinkage and selection operato (LASSO), Support vector machine - recursive feature elimination (SVM-RFE), and Artificial Neural Networks (ANN), has revolutionized biomarker discovery and prognosis modeling, offering precise insights from complex data (9).

In this study, we combined WGCNA and machine learning to analyze TCGA LUAD data, identifying four key immune genes linked to overall survival. We constructed an immune prognostic model, explored their clinical correlations and immune infiltration mechanisms, providing a new basis for LUAD diagnosis, treatment, and prognosis improvement.

## 2 Materials and methods

### 2.1 Research process

**Figure 1:**
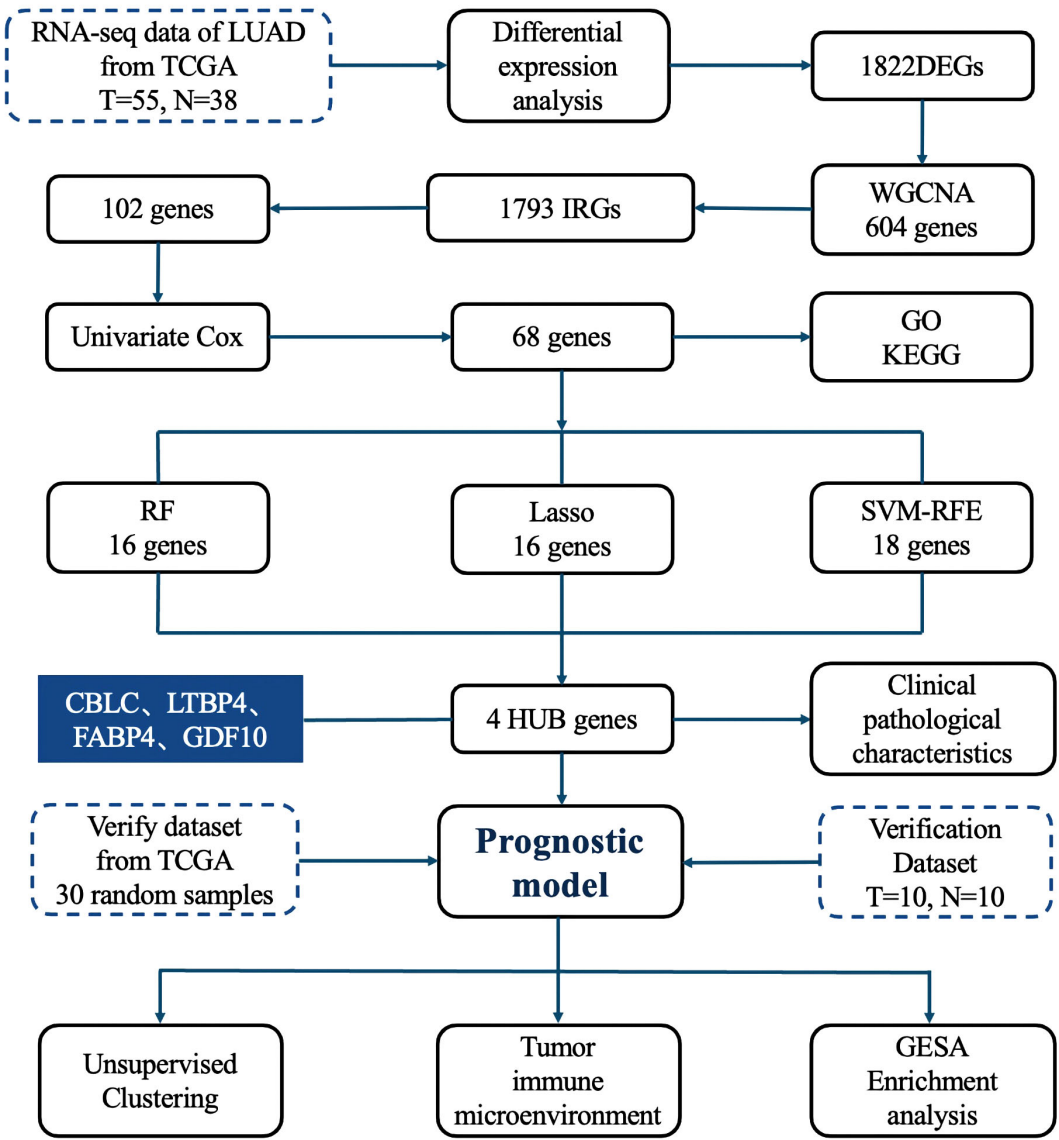
Research process.

The detailed research workflow is illustrated in **Figure 1**.

### 2.2 Data acquisition and processing

Gene expression data for LUAD were obtained from The Cancer Genome Atlas (TCGA) database (https://portal.gdc.cancer.gov/) using the R software package “TCGAbiolinks”.

Clinical data were directly acquired from TCGA, comprising 535 LUAD patients and 59 healthy controls. After rigorous quality control and preprocessing, which included removal of duplicate and incomplete records, we established our training set with 55 LUAD patients and 38 healthy controls. Concurrently, we constructed a TCGA validation set by randomly selecting 30 samples with complete grouping information and unbiased gene expression profiles, excluding those used in the training set.

### 2.3 Clinical sample acquisition

From January 2020 to April 2022, we prospectively collected medical records and tissue specimens from 10 patients undergoing surgical resection for primary LUAD at the First Affiliated Hospital of Wenzhou Medical University. These 10 LUAD specimens and their matched adjacent normal tissues (n = 10) were subsequently processed for gene sequencing analysis, serving as an independent external validation cohort. This study protocol was approved by the Institutional Review Board of the First Affiliated Hospital of Wenzhou Medical University (Approval No. YS2023-706).

### 2.4 RNA preparation and sequencing protocol

Paired-end sequencing (150 bp) was conducted on the Illumina NovaSeq platform (Illumina, California, USA) following standard protocols. Raw sequencing data were processed on the BMK Cloud bioinformatics platform (https://www.biocloud.net) for downstream analysis.

### 2.5 Differential expression gene analysis

In the R software environment, the “limma” package was used to normalize and annotate probe data from a training set of LUAD patients (55 cases) and control population (38 cases) in public databases. Differential gene expression analysis was performed to identify differentially expressed genes (DEGs) between the lung cancer group and the control group. To visualize the expression patterns of these DEGs, hierarchical clustering was conducted using the heatmap package, while the *ggplot2* package was employed to generate volcano plots, providing an intuitive representation of gene expression differences.

### 2.6 Construction of gene co-expression networks

To elucidate the interactions among genes in LUAD, we employed WGCNA on expression profile data from the TCGA training set. Data integrity was assessed using the “Good Samples Genes” function to ensure robust downstream analysis.

### 2.7 Immune-related gene screening

Following the removal of duplicate entries, IRGs were retrieved from the Immunology Database and Analysis Portal (ImmPort, https://www.immport.org), yielding a total of 1,793 unique genes (10). These genes were subsequently cross-referenced with those identified through gene clustering analysis using Venn diagrams, enabling the precise selection of immune-related genes.

### 2.8 Cox analysis related to prognosis

To investigate the association between DEGs and patient overall survival, univariable Cox regression analysis was performed. Hazard ratios (HRs) were calculated to classify genes as either protective (HR < 1) or risk factors (HR > 1) for prognosis. For visual representation of the results, a volcano plot was generated to illustrate the statistical significance and risk ratios of DEGs. Additionally, a forest plot was employed to display detailed univariable Cox regression results, including HR values and their 95% confidence intervals (CIs).

### 2.9 GO enrichment analysis and KEGG pathway analysis

Gene Ontology (GO) term (https://www.geneontology.org/) and Kyoto Encyclopedia of Genes and Genomes (KEGG) pathway (https://www.genome.jp/kegg/) enrichment analyses were conducted using the R package “cluster Profiler” (11).

### 2.10 Machine learning

In this study, we adopted three distinct machine learning algorithms—LASSO, SVM-RFE, and RF—to screen out HUB genes from specific modules. By independently applying these three algorithms and intersecting their selected genes, we identified robust diagnostic biomarkers.

### 2.11 Gene expression difference analysis

To evaluate the differential expression of HUB genes between tumor and normal tissues, we generated box plots using the “ggplot2” package. Statistical significance was assessed using Student’s t-tests. To ensure the robustness of our results, we further validated these findings in an independent external dataset.

### 2.12 Single-gene diagnostic ROC analysis

For the key genes identified during screening and validation, we utilized RNA-Seq data from LUAD, and adjacent non-tumor tissues obtained from the TCGA database. To assess the diagnostic potential of individual genes, we performed logistic regression analysis, treating gene expression levels as the independent variable and LUAD status (tumor vs. non-tumor) as the dependent variable. The diagnostic performance of each gene was quantified by calculating the area under the receiver operating characteristic (ROC) curve (AUC).

### 2.13 Gene expression correlation analysis

We employed Pearson correlation analysis to examine pairwise relationships between key genes, followed by visualization using heatmaps. This approach enables the identification of co-expression patterns and potential functional interactions among genes, providing insights into their regulatory networks.

### 2.14 Analysis of clinical and pathological features

To investigate the potential associations between gene expression levels and clinicopathological characteristics, we performed chi-square tests in this study.

### 2.15 Development of an immune-related prognostic model

We constructed a prognosis model for lung adenocarcinoma patients using univariate and multivariate Cox regression analysis methods. First, univariate Cox regression analysis was used to identify individual variables significantly associated with patient survival time. Subsequently, multivariate Cox regression analysis further evaluated the independent effects of these variables on patient prognosis while considering other potential confounding factors. The risk score was calculated as a linear combination of gene expression levels and corresponding Cox regression coefficients. To facilitate clinical use, we created a nomogram using the “rms” package in R.

Model performance was assessed through: 1. Calibration curves to compare predicted vs. actual survival, validated on an independent dataset; 2. ROC curves via the “pROC” package, with AUC values measuring predictive accuracy across thresholds.

### 2.16 Construction of an artificial neural network diagnostic model

We used the “neuralnet” and “neuralnettools” packages in R to construct an ANN diagnostic model based on hub gene expression. The model architecture included an input layer (hub genes), two hidden layers (with 8 and 3 neurons, respectively), and an output layer representing “normal” and “tumor” classes, with softmax activation. To avoid overfitting, we applied 10-fold cross-validation and used gene weight information during training.

Model performance was assessed using accuracy, precision, recall, F1-score, and AUC in both the training and validation sets. ROC curves were also used to evaluate the discriminative ability of both individual hub genes and the ANN model across the TCGA training set, internal validation set, and external cohort. ROC analysis assessed diagnostic accuracy, with AUC values reflecting its classification ability.

### 2.17 Consensus clustering analysis

To identify potential molecular subgroups in LUAD samples, we applied the ConsensusClusterPlus algorithm (12), which determines the optimal number of clusters (k) by resampling the dataset and evaluating clustering stability.

To validate the robustness of the classification, we used two high-dimensional visualization techniques: UMAP and t-SNE. Additionally, Kaplan-Meier survival analysis was performed to assess the prognostic relevance of the identified subgroups by comparing survival curves across groups.

### 2.18 Analysis of immune cell infiltration

To assess immune infiltration patterns in LUAD, we leveraged the TIMER database (https://cistrome.shinyapps.io/timer) (13). Here, we utilized TIMER to examine correlations between the expression of four candidate genes (CBLC, GDF10, LTBP4, and FABP4) and Tumor immune infiltrating cells (TII Cs) populations, providing insights into their potential immunomodulatory roles in LUAD.

For a more comprehensive evaluation of the tumor immune microenvironment, we applied single-sample gene set enrichment analysis (ssGSEA) (14). Using the GSVA and GSEA Base R packages, we quantified immune infiltration levels in each LUAD sample based on 28 immune-related gene sets curated from the TISIDB (http://cis.hku.hk/TISIDB/) database.

### 2.19 Gene Set Enrichment Analysis

To investigate the transcriptomic characteristics of LUAD tumor samples and identify key biological pathways potentially involved in disease pathogenesis, we performed Gene Set Enrichment Analysis (GSEA). Pathways with a nominal P-value (NOM P) < 0.05 and a false discovery rate (FDR Q-value) < 0.05 were considered statistically significant.

### 2.20 Immunohistochemical staining

The Human Protein Atlas (HPA; https://www.proteinatlas.org/) is a comprehensive online repository that maps the expression and localization of human proteins across various cells, tissues, and organs using diverse biological techniques. We analyzed the immunohistochemical (IHC) staining patterns of selected hub genes in normal lung tissue and lung cancer specimens using data and analytical tools available on the Human Protein Atlas platform.

### 2.21 Statistical analysis

Significant differences between groups were assessed using the log-rank test and univariate COX regression analysis, with P-values and HR with 95% CI calculated. Multivariate COX regression analysis and stratified analysis were performed to evaluate the independence of the risk score model. The performance of gene prognostic signatures was assessed using ROC curves, with the area under the curve (AUC) as the metric. Statistical significance was defined as P < 0.05. All statistical analyses were conducted using R language version 4.3.0.

## 3 Results

### 3.1 Patient information

The original mRNA expression data for LUAD were obtained from TCGA database, comprising 535 LUAD tumor samples and 59 matched adjacent normal tissue samples. Following quality control and data filtering, our final study cohort consisted of 55 LUAD samples as the experimental group and 38 adjacent normal tissue samples as controls. The complete clinical characteristics of the 55 LUAD cases are summarized in **Table 1**.

**Table 1:** Patient clinical characteristics table.

Category		LUAD
Gender	Male	33
	Female	22
T	T1	9
	T2	26
	T3	13
	T4	7
N	N0	33
	N1	12
	N2	10
M	M0	37
	M1	9
	MX	9
Stage	Stage I	14
	Stage II	17
	Stage III	15
	Stage IV	9

### 3.2 Differential analysis

**Figure 2:**
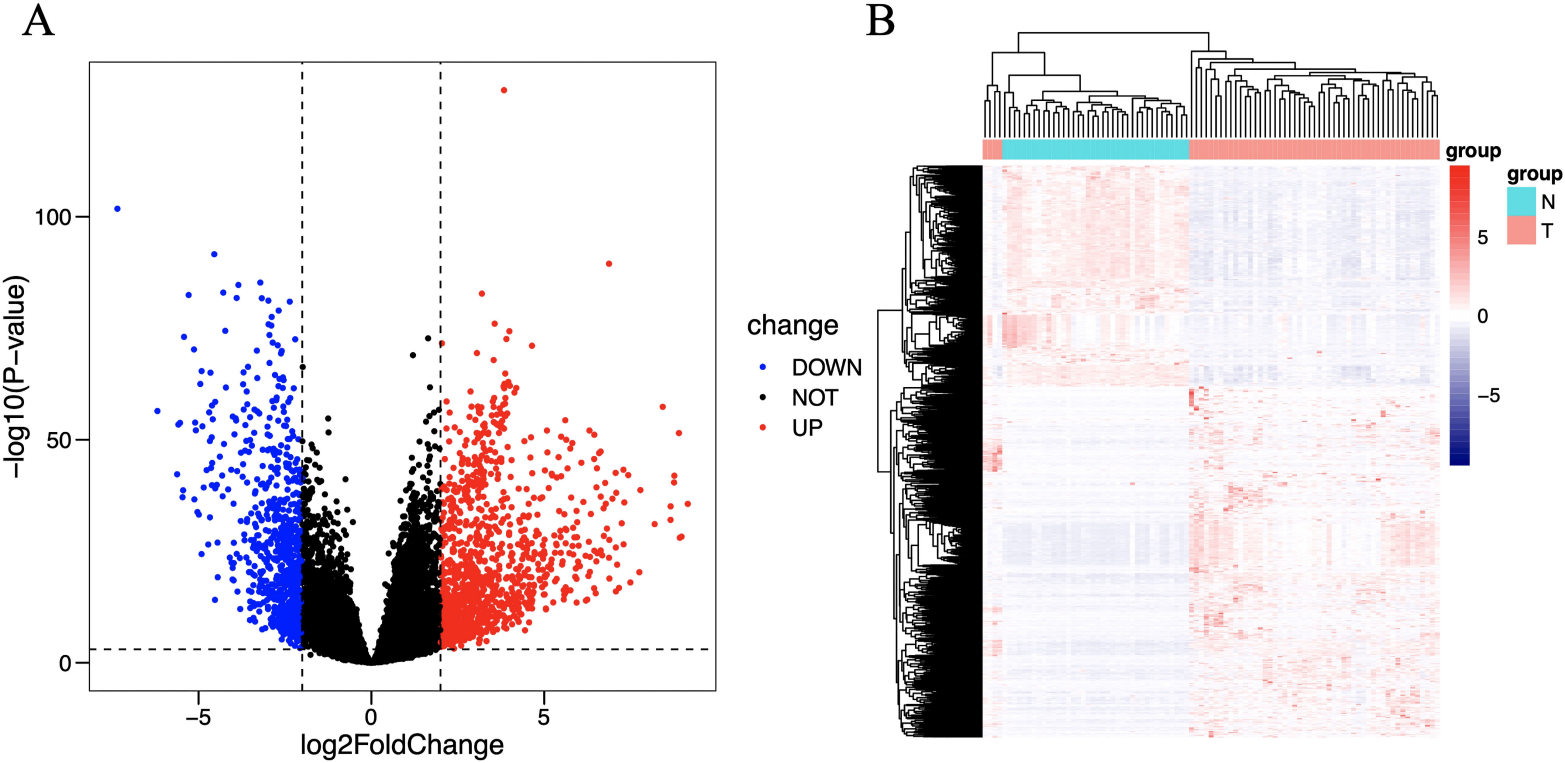
Volcano plot **(A)** and heat map **(B)** of differentially expressed immune genes. The horizontal axis of the heat map represents the samples: the blue area represents normal tissue, and the red area represents tumor tissue; the vertical axis represents genes. On the volcano map, the blue area represents downregulated differentially expressed genes, and the red area represents upregulated differentially expressed genes.

There is a significant difference between the tumor group and the control group. Differential analysis identified a total of 1822 DEGs (log2FC > 2, P < 0.001), including 719 downregulated genes and 1103 upregulated genes. These differences are intuitively displayed in the volcano plot (**Figure 2A**) and heat map (**Figure 2B**).

### 3.3 WGCNA

**Figure 3:**
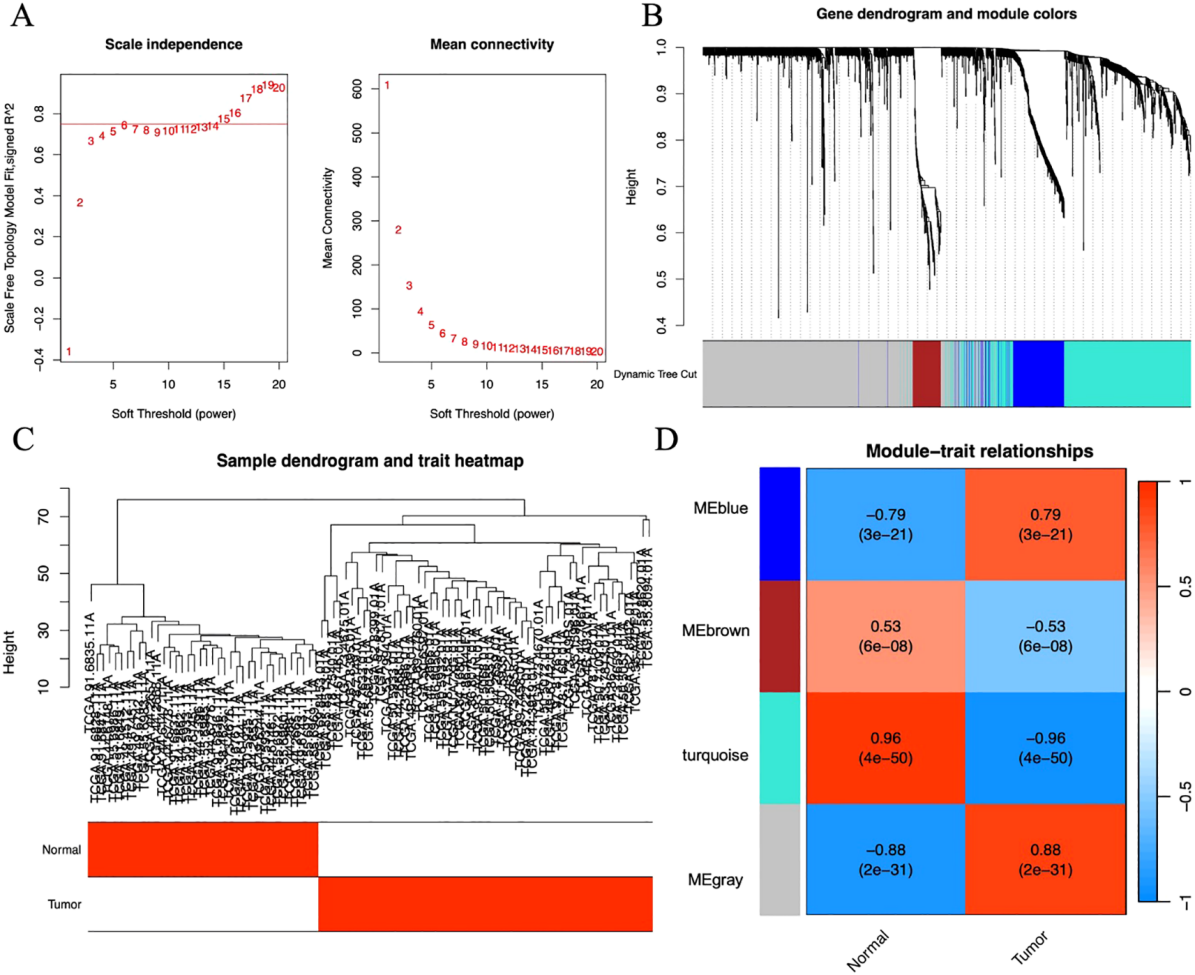
**(A)** Scatter plot of fitting index and average connectivity: The left figure shows the relationship between soft threshold (power) and fitting index, and the right figure shows the relationship between soft threshold and average connectivity. To achieve a correlation of 0.9, β=17 is selected as the optimal soft threshold. **(B)** Sample clustering diagram and trait heat map: Show the results of sample clustering and grouping information of tumor and normal samples, with red representing the tumor group and white representing the normal group. **(C)** Module clustering tree diagram: Construct a scale-free network and topological overlap matrix (TOM), and perform hierarchical clustering based on the hclust function. Set the minimum number of module genes to 100, depth split=2, and module merging threshold to 0.25 (merge when similarity > 0.75). **(D)** Module and clinical trait correlation heat map: The horizontal axis represents traits (tumor and control), and the vertical axis represents each module. The blue module with the highest correlation with the tumor group (Cor=0.96, P=4e-50) is selected for subsequent analysis.

We adopted the WGCNA method to identify gene modules associated with LUAD. First, by evaluating the scale-free fit index and average connectivity under different soft thresholds, the optimal soft threshold was determined to be β=17 (**Figure 3A**). Subsequently, sample clustering analysis was performed (**Figure 3B**), a topological overlap matrix was constructed, and hierarchical clustering was applied to identify modules. By using the dynamic tree cut algorithm and merging modules with similarity greater than 0.75, a final module clustering map was obtained (**Figure 3C**). Then, correlation analysis between modules and clinical features was conducted, and a heatmap was drawn to show the correlation coefficients and P-values (**Figure 3D**). A total of 4 modules were identified, among which the blue module with the highest correlation with the tumor group (r=0.96, P=4e-50) was selected, containing 604 genes.

### 3.4 Immune gene screening

**Figure 4:**
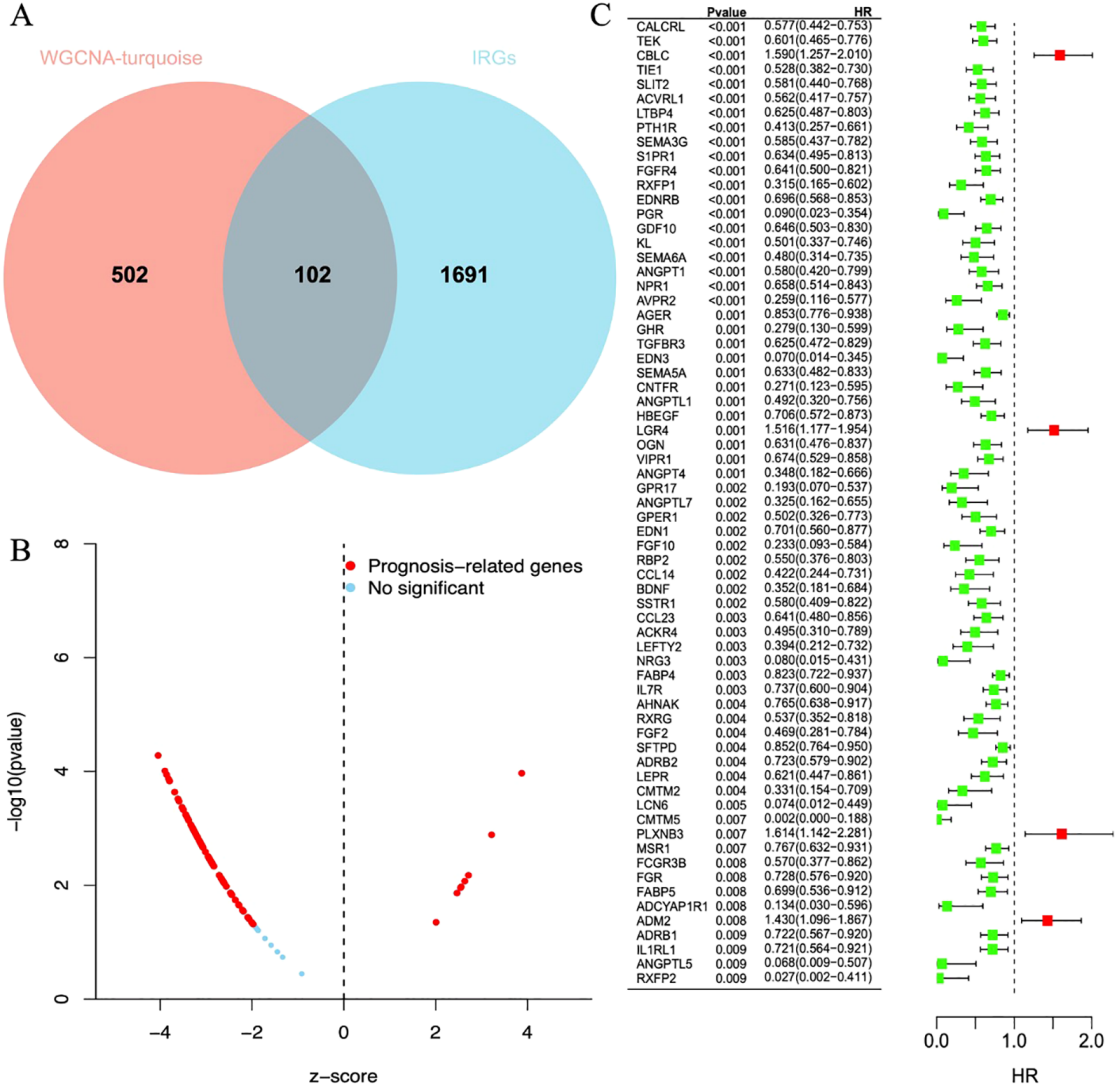
**(A)** The Venn diagram shows reliable biomarkers between WGCNA and IRGs. **(B)** Volcano plot of prognostic genes. Red indicates genes related to prognosis, and blue indicates genes without significant prognostic correlation. **(C)** Forest plot of 68 differentially expressed immune genes in a single-factor COX regression model. The brackets indicate the 95% confidence intervals. HR stands for hazard ratio, with HR < 1 displayed in green, indicating a risk factor; HR > 1 displayed in red, indicating a protective factor.

A set of data consisting of 1793 IRGs was obtained from IMMPORT database (15). To further explore the immune genes associated with LUAD, we compared these immune genes with 604 genes screened by gene clustering methods. A total of 102 immune genes closely associated with LUAD were identified, and these findings were visualized in a Venn diagram (**Figure 4A**).

### 3.5 Prognostic analysis

After a one-way regression analysis of 102 differential immune genes related to LUAD, the results showed that 68 differential immune genes were significantly correlated with the overall survival rate of LUAD patients (P < 0.05) (**Figures 4B,C**). These significantly related differential immune genes may affect tumor progression and patient survival prognosis by affecting the immune microenvironment of LUAD.

### 3.6 Enrichment analysis

**Figure 5:**
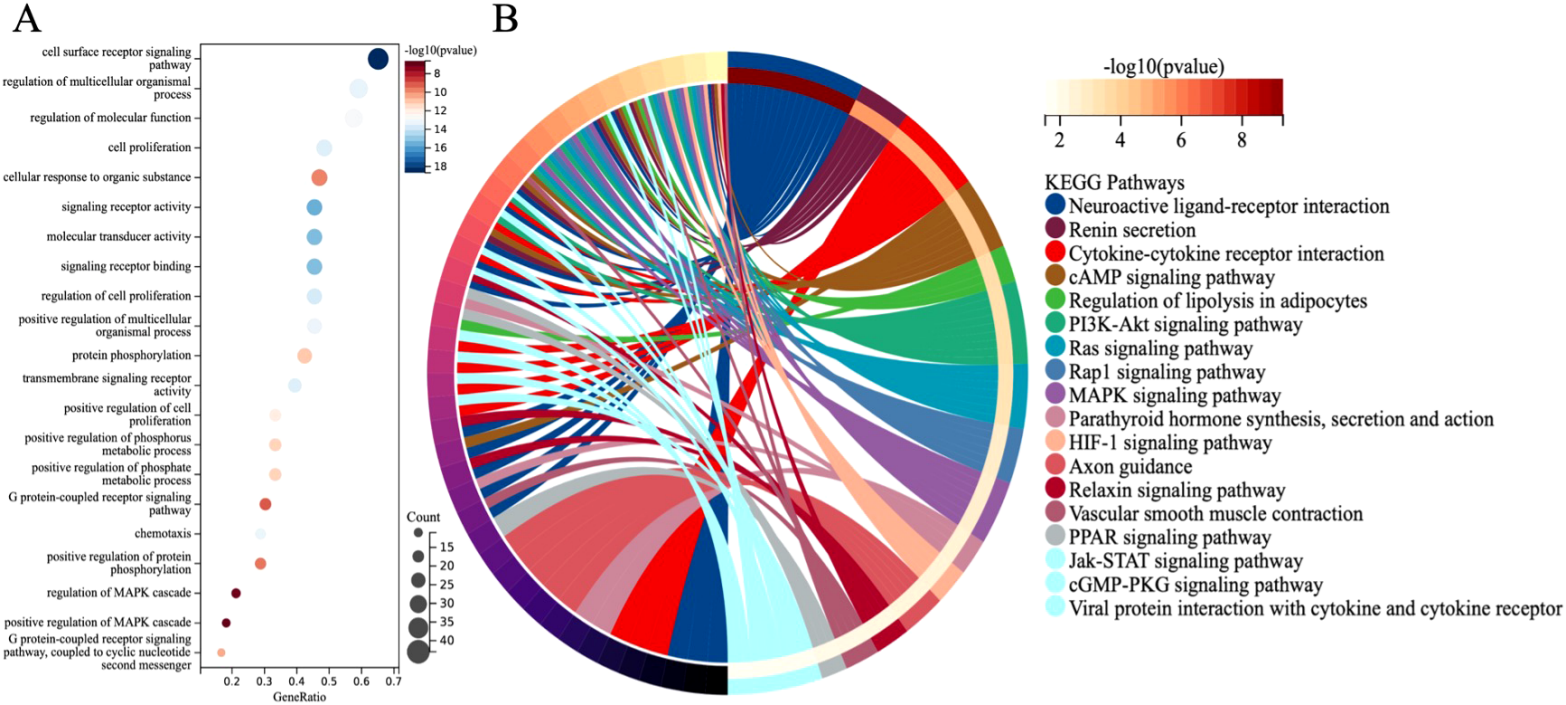
**(A)** GO enrichment analysis. **(B)** KEGG major pathway enrichment analysis.

GO and KEGG analyses revealed the functional localization of 68 differentially expressed immune genes significantly associated with overall survival in lung adenocarcinoma. GO analysis indicated that these genes are mainly involved in signal pathway regulation (such as cell surface receptor signaling, G protein-coupled receptor pathway), cell proliferation, substance metabolism (such as protein phosphorylation), and immune inflammatory response (such as chemotaxis, MAPK cascade regulation) (**Figure 5A**). KEGG analysis further found that these genes are enriched in immune-related pathways (such as PI3K-Akt and Jak-STAT pathways) and tumor proliferation-related pathways (such as Ras, Rap1, and MAPK signaling pathways) (**Figure 5B**). These results help to better understand the role of differentially expressed immune genes in the occurrence and development of LUAD.

### 3.7 Identification of diagnostic biomarkers

**Figure 6:**
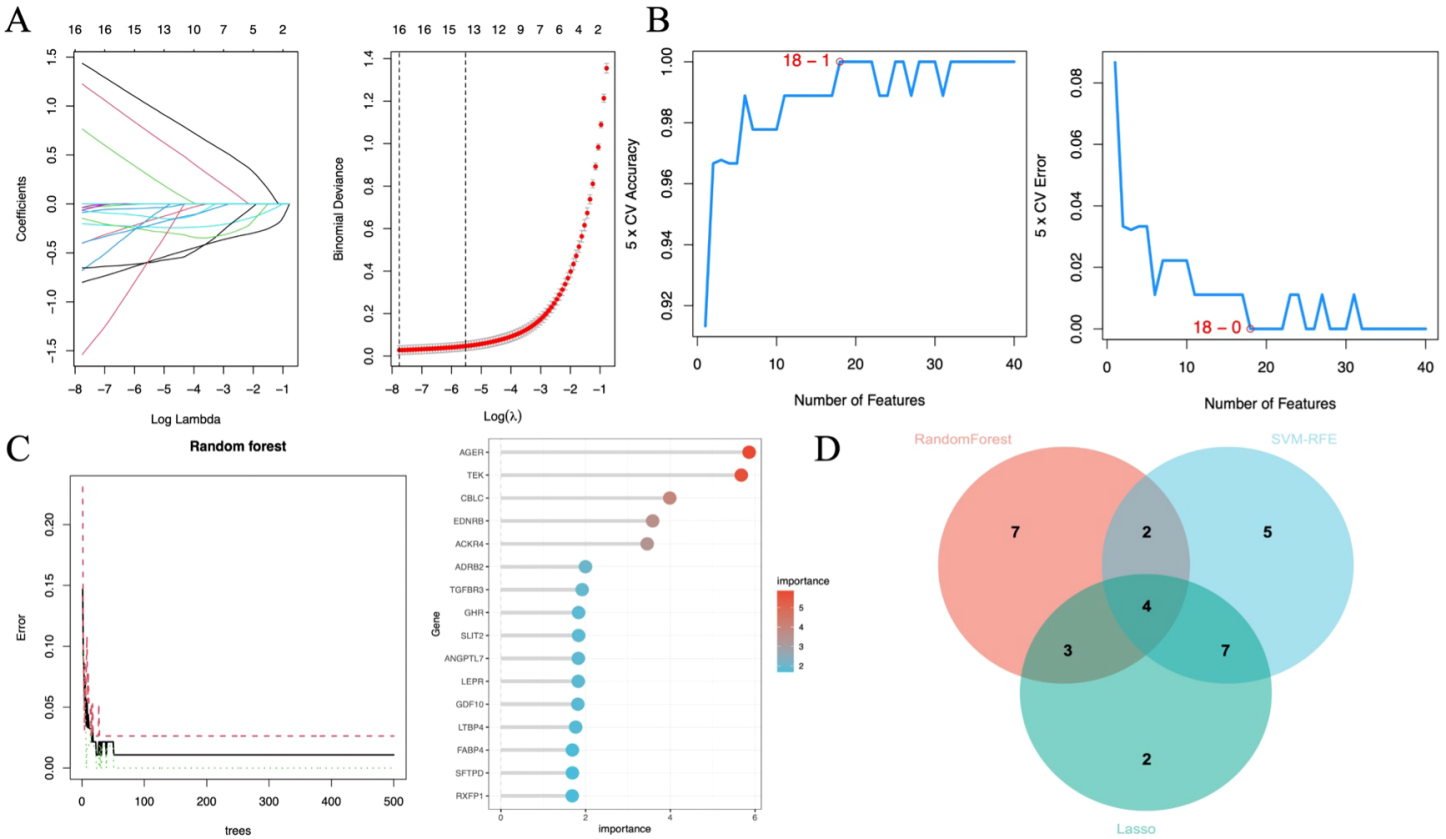
Diagnostic biomarker screening based on machine learning. **(A)** Variable selection in the LASSO model (n=3). **(B)** SVF-RFE algorithm screens the best biomarkers (n=6). **(C)** Significant features selected by the random forest algorithm (n=7). **(D)** Venn diagram of overlapping genes in the three algorithms.

Machine learning identified 68 candidate genes. LASSO, SVM-RFE, and RF algorithms further narrowed them to 16, 18, and 16 genes, respectively. By overlapping the three groups, four key diagnostic biomarkers were finally identified (**Figure 6**). The information related to 4 genes is shown in **Table 2**.

**Table 2:** Overall information of the 4 genes constructing prognostic features.

Gene ID	Gene symbol	Gene type	Chromosome	Gene start point (bp)	Gene end (bp)
ENSG00000170323	FABP4	Protein encoding	chr8	81478419	81483236
ENSG00000266524	GDF10	Protein encoding	chr10	47300197	47313577
ENSG00000090006	LTBP4	Protein encoding	chr19	40592883	40629820
ENSG00000142273	CBLC	Protein encoding	chr19	44777869	44800652

### 3.8 Diagnostic biomarker verification

**Figure 7:**
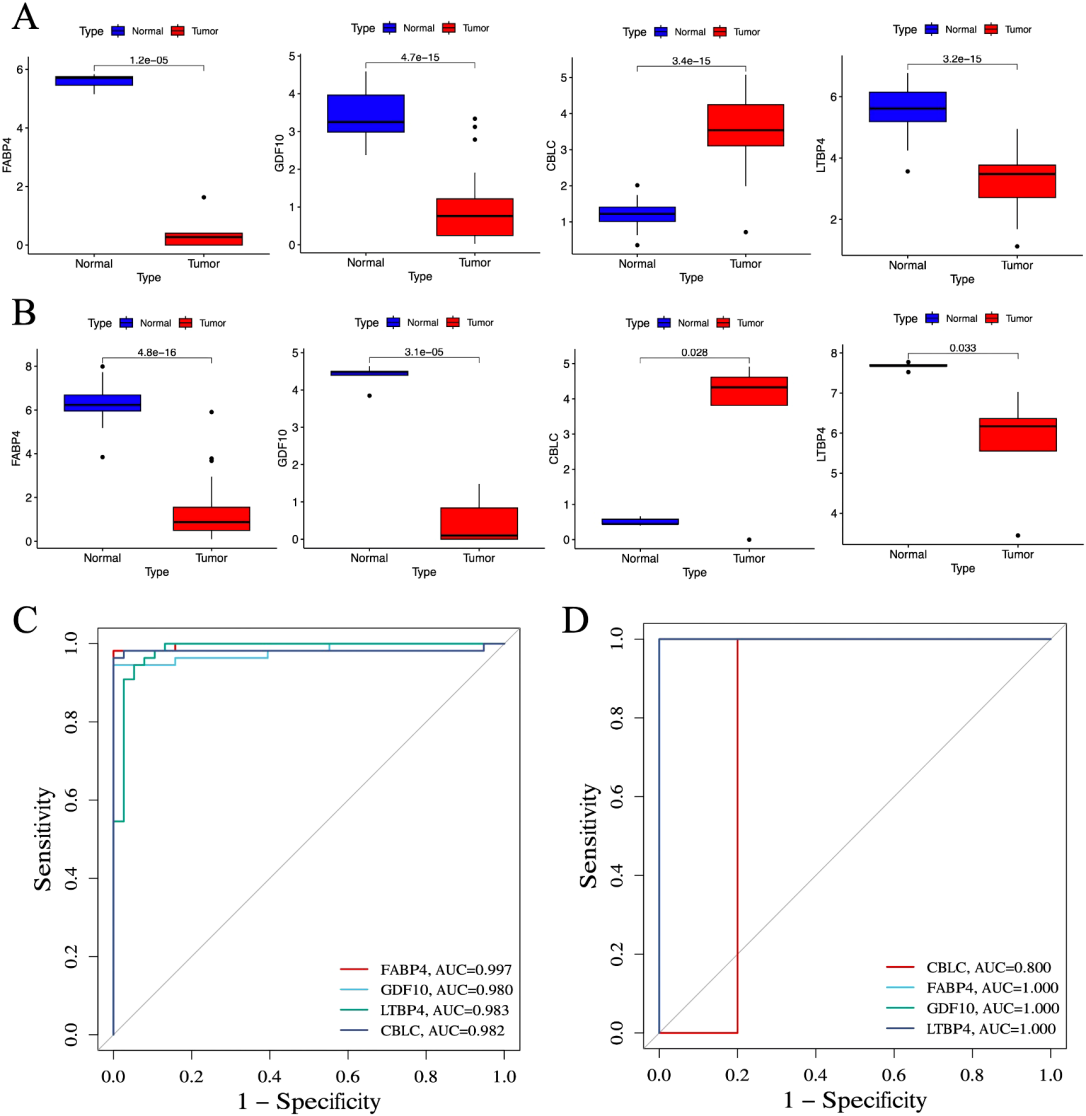
Verification of identified biomarkers. **(A)** Box plot for differential expression analysis in TCGA cohort (n =55). **(B)** Box plot for differential expression analysis in independent external sequencing cohorts (n =10). **(C)** ROC curve for evaluating the diagnostic ability of the TCGA cohort. **(D)** ROC curve for evaluating the diagnostic ability of the independent external sequencing cohort. P < 0.05 is considered statistically significant.

In lung cancer research, by comparing the lung cancer samples in the TCGA cohort with the normal control group, it was found that the expression level of CBLC genes was significantly increased, while the expression levels of the three genes FABP4, GDF10, and LTBP4 were significantly decreased (**Figure 7A**). This result was validated in an independent externally sequencing sample cohort (**Figure 7B**), showing gene expression patterns consistent with the TCGA cohort, enhancing the reliability and universality of this finding.

To evaluate the predictive utility of these genes as biomarkers of lung cancer, the study performed a ROC curve analysis. In the TCGA cohort, the AUC values ​​of CBLC, FABP4, GDF10, and LTBP4 genes were 0.982, 0.997, 0.980, and 0.983, respectively (**Figure 7C**), indicating that these genes have a high diagnostic efficiency in distinguishing lung cancer samples from normal control groups. In an independent external sequencing sample cohort, these genes also performed impressively, with the AUC value for CBLC at 0.800, while the AUC values for FABP4, GDF10, and LTBP4 all reached 1.000 (**Figure 7D**), further validating their potential as diagnostic markers for lung cancer.

### 3.9 Analysis of clinical staging and genetic correlation

**Figure 8:**
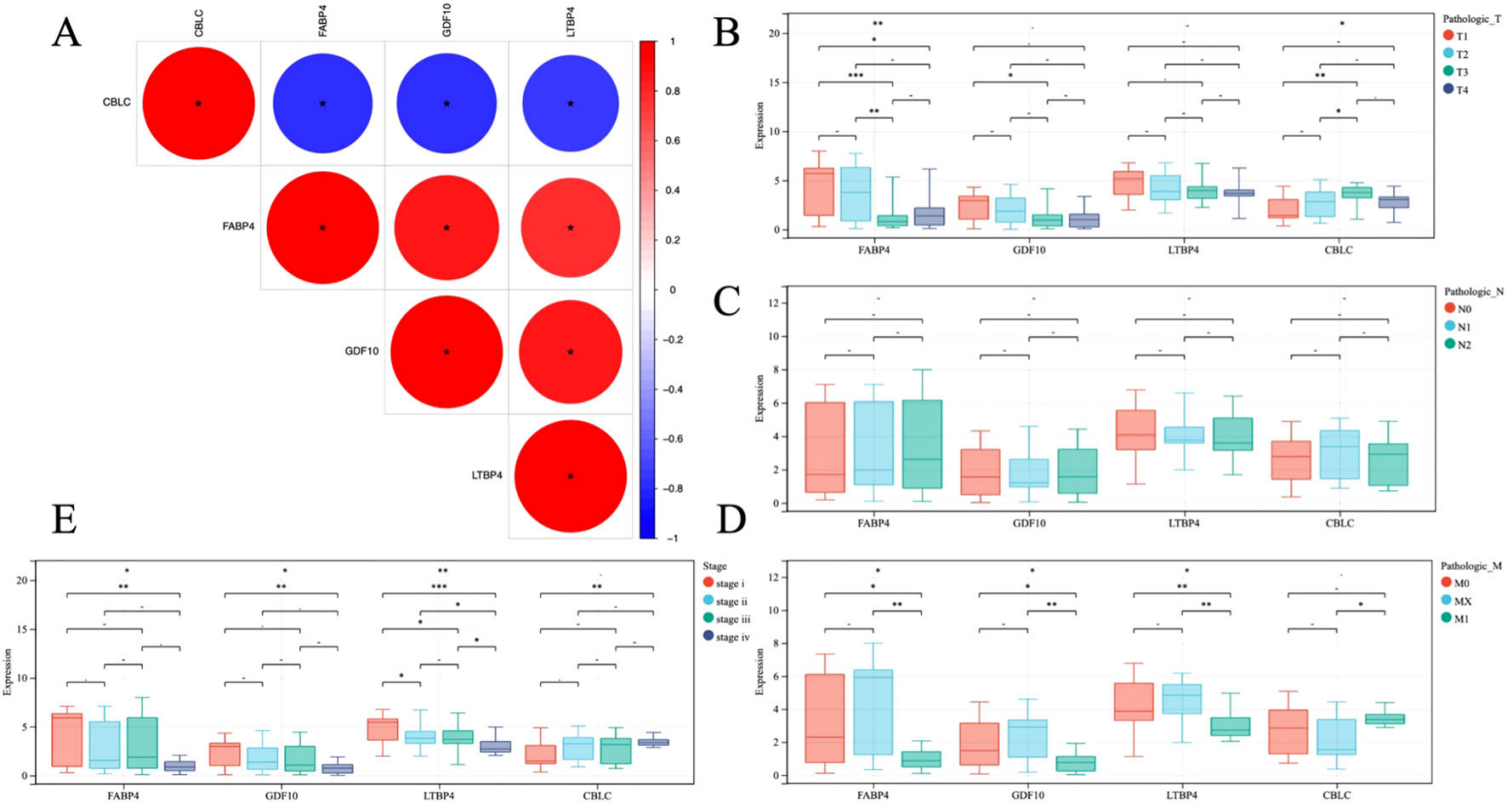
**(A)** Heat map of gene correlation analysis, blue and red indicate positive and negative correlation, respectively. **(B–E)** Analysis of correlation between gene and clinical stage. * P < 0.05, **P < 0.01, *** P < 0.001.

To gain a deeper understanding the role of the four genes CBLC, FABP4, GDF10, and LTBP4, and their genetic interactions in LUAD, Pearson correlation coefficients were calculated. The calculation results (**Figure 8A**) show that, except for CBLC, there is a good correlation between the three genes FABP4, GDF10, and LTBP4, indicating that they may play similar or synergistic roles in the development of LUAD.

In addition, through statistical analysis of the TCGA dataset, the study further explored the relationship between these gene expression levels and the clinicopathological status of patients with LUAD (**Figures 8B–E**). The analysis results revealed, the high expression of CBLC in LUAD is positively correlated with poor clinical and pathological stages in patients, which means that the high expression of CBLC may be an indicator of poor progression and prognosis of lung adenocarcinoma. In contrast, high expression of FABP4, GDF10, and LTBP4 is associated with better clinical and pathological stages, indicating that the increased expression levels of these genes may be related to a better prognosis of LUAD.

### 3.10 Establishment of immune prognostic model

**Figure 9:**
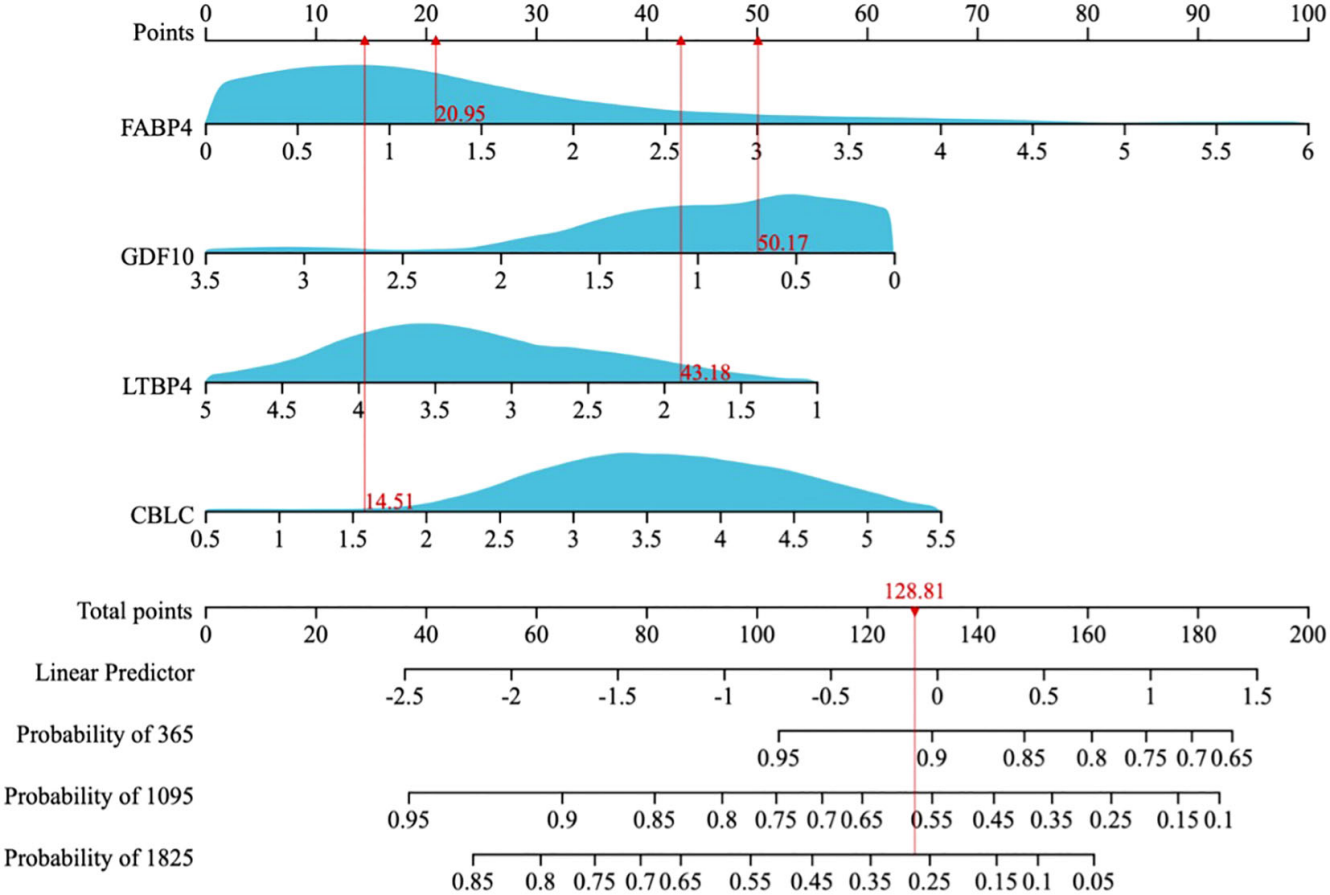
A nomogram for predicting the 1-year, 3-year, and 5-year overall survival (OS) probabilities in LUAD patients.

**Figure 10:**
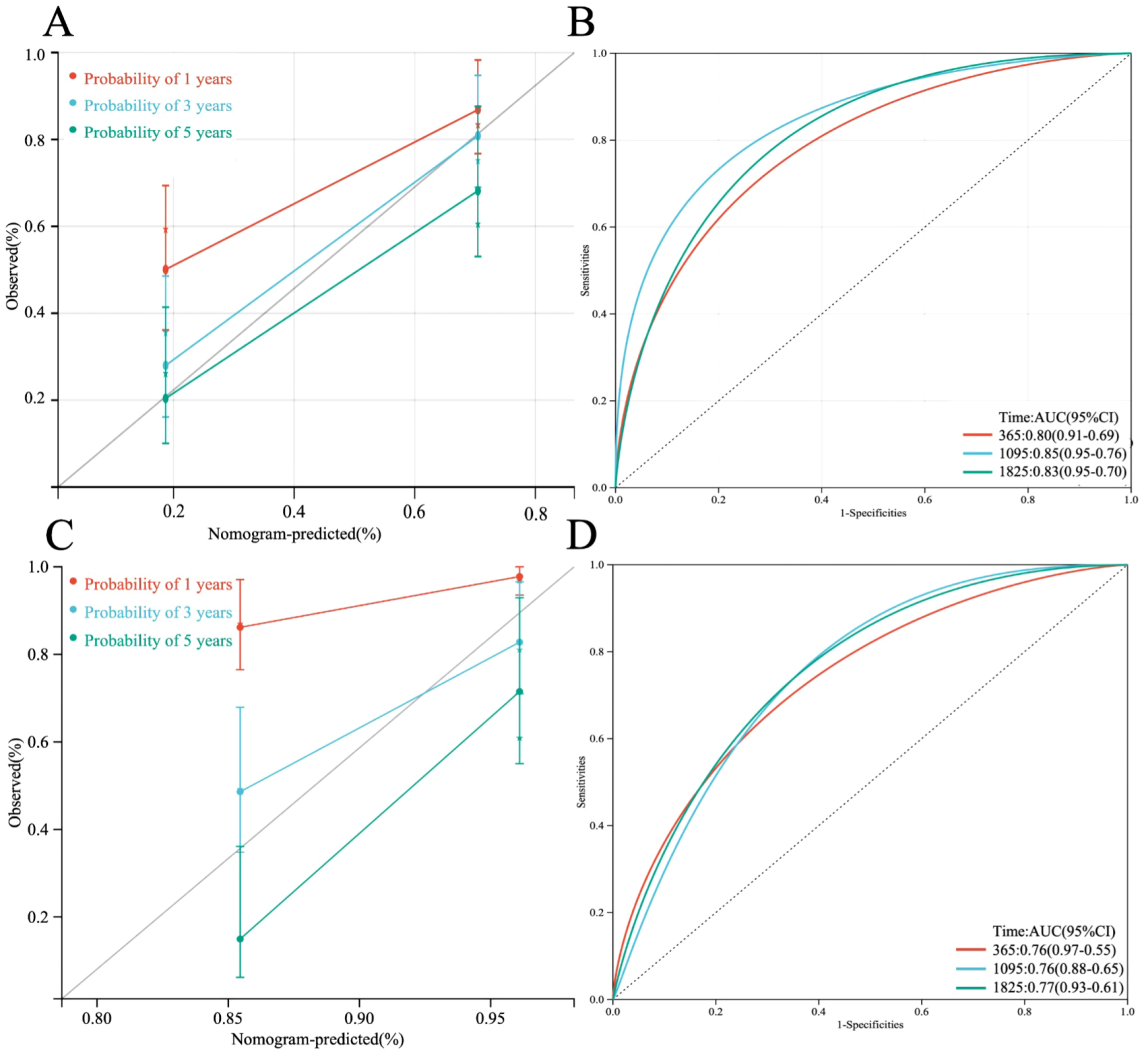
**(A)** Calibration plot of the line chart for the training group, used to predict the probability of OS at 1, 3, and 5 years. **(B)** Time-dependent ROC curve analysis of the immunological prognostic model line chart for the training group. **(C)** Calibration plot of the line chart for the TCGA validation group, used to predict the probability of OS at 1, 3, and 5 years. **(D)** Time-dependent ROC curve analysis of the immunological prognostic model line chart for the TCGA validation group.

A multi-factor COX regression analysis was used to construct an immune prognostic model for predicting the 1-year, 3-year, and 5-year survival rates of LUAD patients, which was visualized as a nomogram (**Figure 9**). The analysis results of the calibration curve (**Figures 10A, C**) showed that there is a good consistency between the model predictions and the ideal model. In addition, the AUCvalues of the model training set were 0.80 (95% CI: 0.91–0.69) for 1-year survival rate, 0.85 (95% CI: 0.95–0.76) for 3-year survival rate, and 0.83 (95% CI: 0.95–0.70)for 5-year survival rate, respectively (**Figure 10B**), while in the TCGA validation set, the AUC values were 0.76 (95% CI: 0.97–0.55), 0.76 (95% CI: 0.88–0.65), and 0.77 (95% CI: 0.93–0.61), respectively (**Figure 10D**).

### 3.11 Validation of the immunoprognostic model

**Figure 11:**
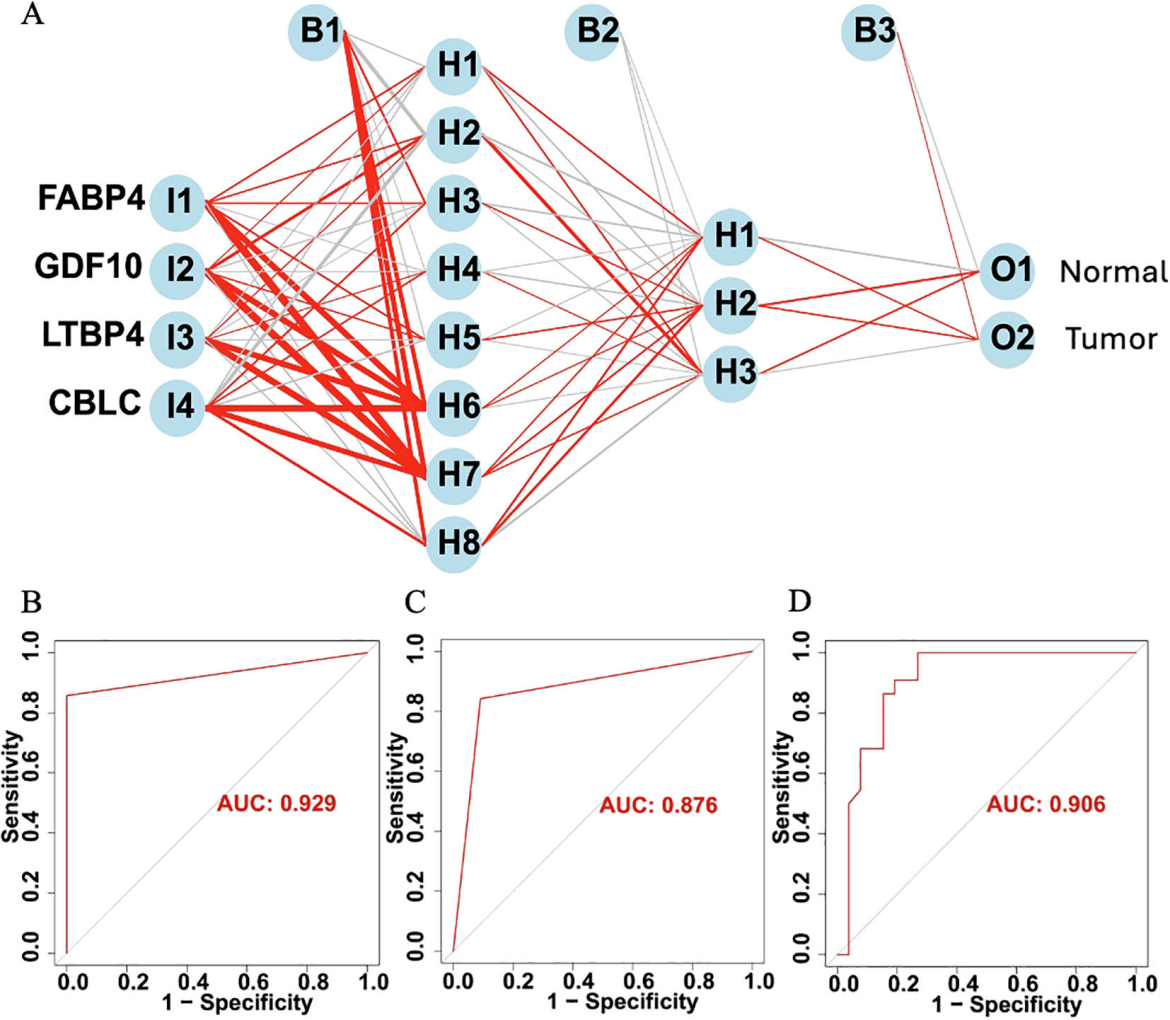
**(A)** Schematic diagram of the ANN model. **(B)** ROC curve of the ANN model in the training set. **(C)** ROC curve of the ANN model in the TCGA validation set. **(D)** ROC curve of the ANN model in the independent external validation set.

Furthermore, based on four HUB genes, an ANN model was developed (**Figure 11A**) to enhance prediction accuracy. The TCGA dataset was used as the training set, while independent sequencing results served as the test set. The diagnostic performance of the ANN model was evaluated using ROC curves. In the training set, the model achieved an AUC of 0.929 (95% CI: 0.878–0.980) (**Figure 11B**), while in the TCGA validation set and independent external validation set, the AUC values were 0.876 (95% CI: 0.812–0.940) and 0.906 (95% CI: 0.849–0.963), respectively (**Figures 11C, D**).

### 3.12 Immune prognostic model subtype analysis

**Figure 12:**
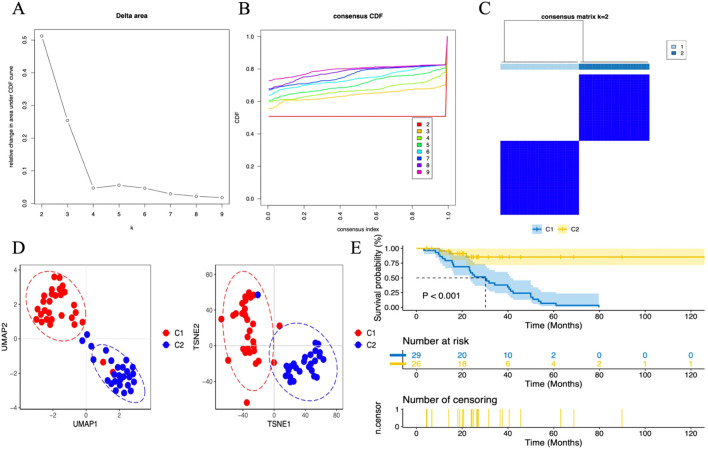
Unsupervised consensus clustering in the TCGA cohort. **(A)** Delta plot showing the change in the area under the consensus clustering CDF curve for k = 2 to 9. **(B)** Cumulative distribution function (CDF) for k = 2 to 9. **(C)** Heatmap displaying two DN sample clusters at k = 2. **(D)** Dimensionality reduction based on consensus clustering results, with UMAP on the left and t-SNE on the right. **(E)** Survival analysis of C1 and C2 subgroups.

When analyzing the gene expression profiles of 55 tumor samples in the TCGA training set cohort, we first performed cluster analysis using the consensus clustering method. The analysis determined that when k=2, the classification of the samples was both highly reliable and stable (**Figures 12A–C**). To further validate the differences between these two subgroups, two dimensionality reduction techniques, Uniform Manifold Approximation and Projection (UMAP) and t-Distributed Stochastic Neighbor Embedding (t-SNE), were employed. The results confirmed the significant differences between the two subgroups (**Figure 12D**). Based on this classification, the samples were divided into two clusters: Cluster 1 (C1, N=29) and Cluster 2 (C2, N=26). Next, survival analysis was performed to explore the prognostic differences between these two subgroups. The analysis results showed (**Figure 12E**) that there were significant survival differences between the two subgroups (P < 0.001).

### 3.13 Immune cell infiltration

**Figure 13:**
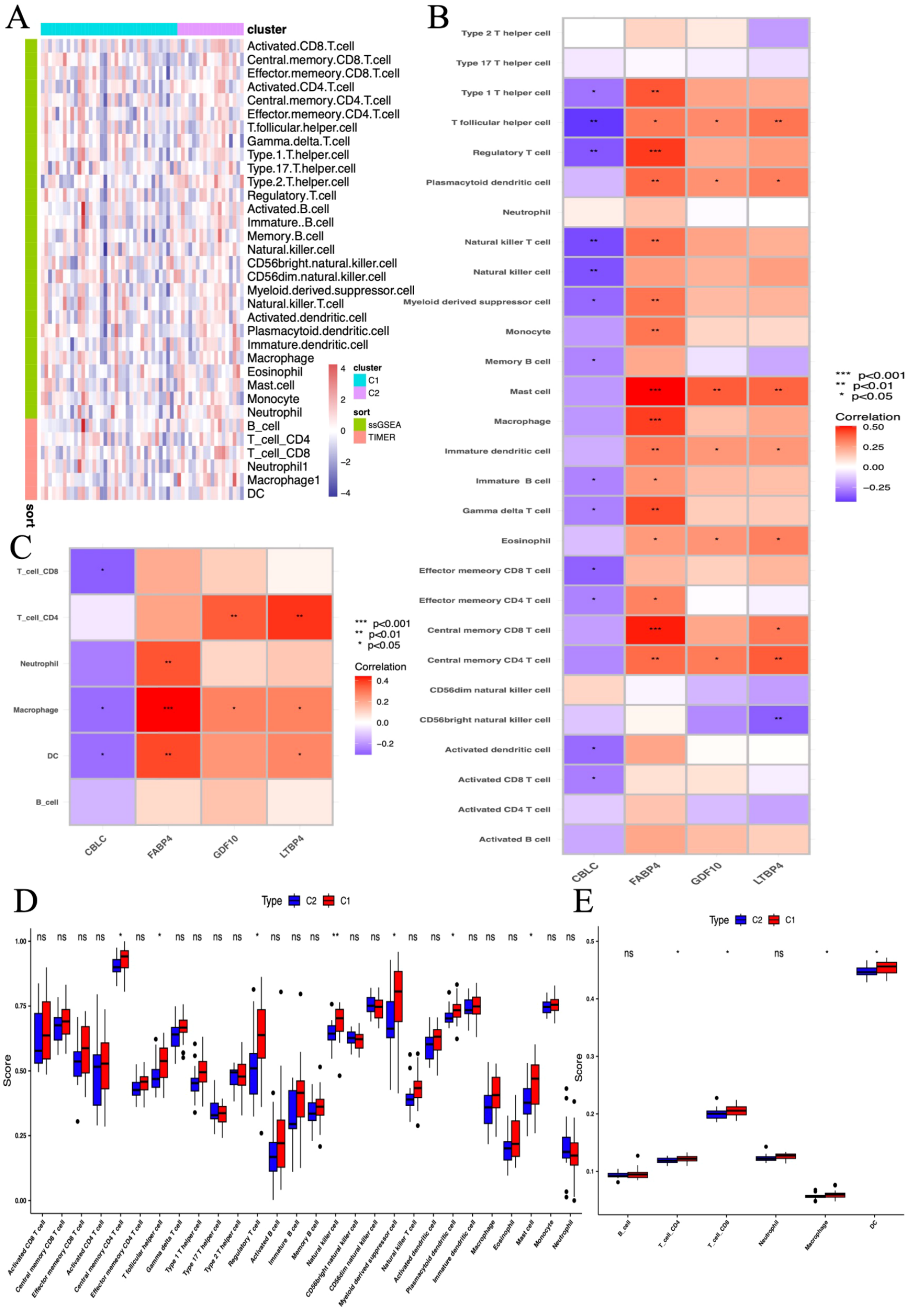
The association between biomarkers and immune infiltration in samples. **(A)** Heatmap of metabolic and immune-related gene sets from GSVA. **(B, C)** Heatmap of immune landscape from ssGSEA. **(D, E)** Box plots showing the correlation between immune subgroups and infiltrating immune cells. *P < 0.05, **P < 0.01, *** P < 0.001.

This study utilized the ssGSEA algorithm to analyze the immune cell infiltration and immune gene correlation in LUAD samples and the control group. The results show (**Figure 13B**): CBLC is negatively correlated with various immune cells (such as activated CD8 T cells, dendritic cells, regulatory T cells, etc.); FABP4 is positively correlated with various immune cells (such as memory T cells, macrophages, neutrophils, etc.); GDF10 is positively correlated with memory CD4 T cells, eosinophils, plasma cell-like dendritic cells, etc.; LTBP4 is positively correlated with memory T cells, eosinophils, and dendritic cells. Further analysis (**Figure 13C**) found that CBLC is mainly negatively correlated with dendritic cells, macrophages, and CD8 T cells; FABP4 is positively correlated with dendritic cells, macrophages, and neutrophils; GDF10 and LTBP4 are mainly positively correlated with macrophages and CD4 T cells, and LTBP4 is also positively correlated with dendritic cells. These results indicate that different genes play different roles in immune cell infiltration, reflecting the complexity and diversity of the LUAD tumor microenvironment.

Further research compared the differences in 28 immune cell types between two immune subgroups and found significant differences and heterogeneity in immune cell infiltration between the high-risk and low-risk groups (**Figure 13A**). Compared to the C2 group, the C1 group had significantly elevated levels of central memory CD4 T cells, follicular helper T cells, regulatory T cells, natural killer cells, myeloid-derived suppressor cells, plasmacytoid dendritic cells, and mast cells (**Figure 13D**). In further studies, we found significant increases in CD4 T cells, CD8 T cells, macrophages, and dendritic cells in the C1 group (**Figure 13E**).

### 3.14 Immunohistochemical staining of HUB genes in normal and tumor tissues

**Figure 14:**
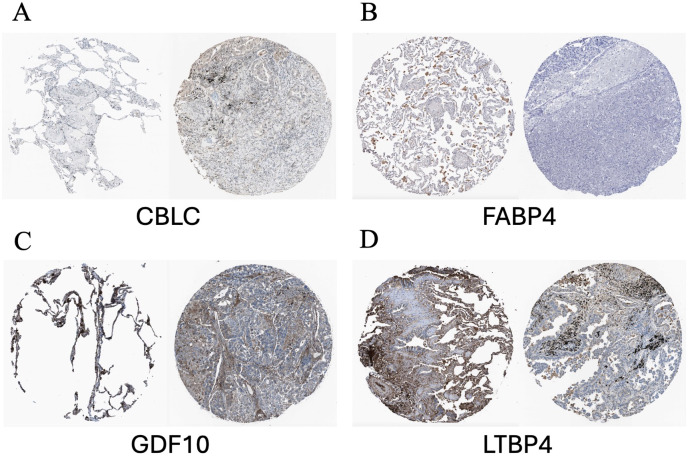
Representative immunohistochemical images of HUB genes in lung adenocarcinoma and normal lung tissues from the HPA database. **(A)** Comparison of CBLC; **(B)** Comparison of FABP4; **(C)** Comparison of GDF10; **(D)** Comparison of LTBP4. HPA, Human Protein Atlas. In each pair of images, the staining on the left side shows the gene expression in normal lung tissue, while the staining on the right side shows the gene expression in lung cancer samples.

According to the immune staining intensity comparisons provided by the HPA database (**Figure 14**), the expression levels of FABP4, GDF10, and LTBP4 were higher in normal samples, while the expression level of CBLC was higher in lung adenocarcinoma samples. This result is consistent with previous studies on gene expression differences.

### 3.15 GESA

**Figure 15:**
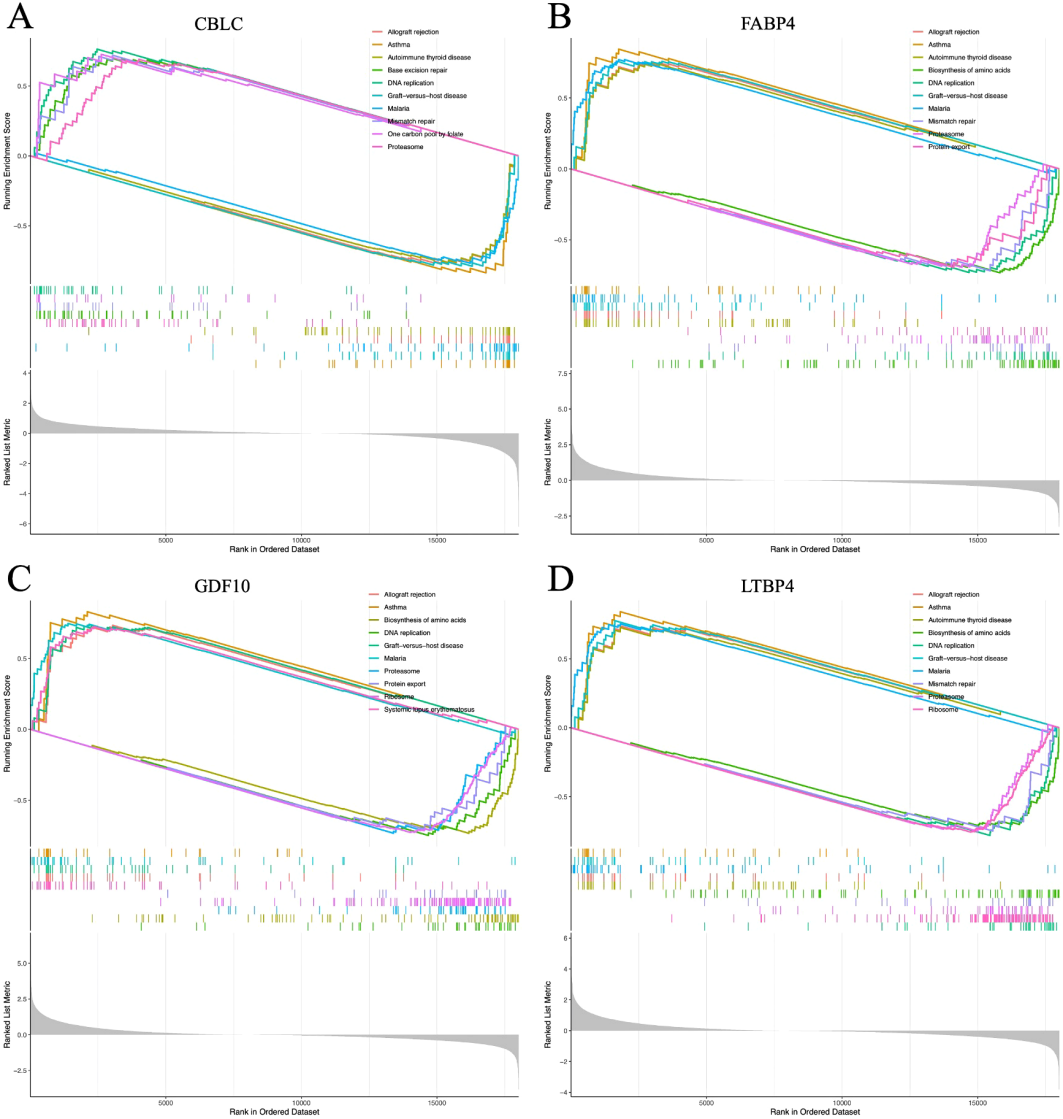
**(A–D)** represent the GSEA enrichment plots for CBLC, FABP4, GDF10, and LTBP4, respectively.

The GSEA analysis of four HUB genes in the progression of lung adenocarcinoma (LUAD) indicates that these genes are mainly enriched in the following biological processes and pathways (**Figure 15**): 1. Allogeneic rejection response 2. Asthma 3. Amino acid biosynthesis 4. DNA replication.

## 4 Discussion

LUAD, a major subtype of NSCLC, remains a global health challenge due to its high incidence, mortality, and poor five-year survival rates despite advances in targeted therapies (16). Traditional prognostic assessments based on clinical and pathological features are limited by interindividual heterogeneity, highlighting the need for molecular biomarkers to guide diagnosis and treatment (17). With the rise of sequencing technologies, precision medicine has become central in LUAD management, promoting individualized therapeutic strategies. Increasing evidence suggests that tumor progression is influenced not only by genetic changes but also by immune system involvement (18). Immune cells play crucial roles in all stages of cancer development (19, 20).

In LUAD, transcriptomic analysis of DEGs highlights enrichment in key biological processes such as signal transduction, cell proliferation, metabolism, immune responses, and inflammation. KEGG analysis further implicates immune-related pathways, notably PI3K/Akt and Jak-STAT, in LUAD progression. The PI3K/Akt axis promotes tumor survival and proliferation (21), while Jak-STAT facilitates immune evasion (22), with their interplay potentially enhancing tumor resilience. These findings underscore the functional relevance of DEGs and reveal therapeutic opportunities to simultaneously inhibit tumor growth and immune escape, improving LUAD outcomes.

WGCNA is a robust bioinformatics tool designed to identify functionally related gene modules associated with specific biological states or disease prognosis from large-scale gene expression datasets (23, 24). In LUAD studies, WGCNA combined with immune-related genes identified four key hub genes: CBLC, FABP4, GDF10, and LTBP4, showing significant differential expression between tumor and normal samples. CBLC is significantly upregulated in LUAD, especially in patients with poor prognosis, suggesting that it may promote tumor invasion (25, 26). Conversely, FABP4, GDF10, and LTBP4 are expressed at higher levels in normal tissue and patients with good prognosis, possibly having antitumor or immune protective effects. CBLC belongs to the CBL family and is an E3 ubiquitin ligase involved in EGFR ubiquitination and degradation, widely upregulated in various cancers, including NSCLC, hinting at its oncogenic potential (27–29). FABP4 regulates lipid signaling and metabolism, and its high expression in lung cancer is associated with poor prognosis (30–34)​​. LTBP4 is a member of the TGF-β pathway, which can both inhibit tumor growth and promote progression in tumors, and its downregulation is common in advanced LUAD (35–39). GDF10 is involved in antitumor and Epithelial-mesenchymal transition (EMT) (40, 41)​​, and is often associated with methylation dysregulation in lung cancer, having potential as a therapeutic target (42, 43). In summary, these four genes play an important role in the occurrence and development of LUAD and are expected to become potential therapeutic targets and prognostic biomarkers.

Nomograms are valuable tools for integrating clinical features to predict patient outcomes. Here, we constructed a nomogram combining clinical parameters and immune-related signatures to estimate Overall survival (OS) in LUAD patients. Calibration plots showed strong agreement between predicted and actual survival, confirming the model’s reliability.

ROC analysis assessed predictive accuracy, with AUCs of 0.83 (training set) and 0.76 (TCGA validation set), indicating strong discrimination. An ANN model further improved prognostic precision, showing consistent performance across TCGA and external datasets, confirming the robustness of our immune-based LUAD prognostic framework.

Our findings further demonstrated a strong association between the identified hub genes and key biological processes, including immune response, inflammation, cell proliferation, and the regulation of cytokines and chemokines. Using ssGSEA, we characterized the immune cell infiltration landscape in the LUAD microenvironment, revealing significantly elevated levels of CD4+ T cells, CD8+ T cells, macrophages, and dendritic cells in LUAD tissues compared to normal controls. Notably, CD4+ and CD8+ T cells are well-established for their potent antitumor activity (44, 45), while dendritic cells and macrophages play critical immunomodulatory roles across multiple cancer types (46–48). Correlation analyses indicated that most hub genes exhibited positive associations with these immune cell populations—particularly CD4+ T cells, CD8+ T cells, macrophages, and dendritic cells—with the notable exception of CBLC, which showed negative correlations. This observation aligns with the poor prognostic implications of elevated CBLC expression in LUAD, collectively underscoring the pivotal role of the immune microenvironment in LUAD pathogenesis and clinical outcomes (49–51). Our results further suggest that the four hub genes (CBLC, FABP4, GDF10, and LTBP4) may serve as key regulators of LUAD progression.

Despite providing valuable insights, this study has limitations. Large, multicenter clinical validations are needed, and reliance on TCGA RNA-seq data may limit generalizability. Moreover, the lack of functional validation calls for cautious interpretation of the bioinformatic results.

Although this study provides valuable insights, it has some limitations. First, to verify the accuracy of the predictive model, more large-scale evidence-based medical studies from different centers are needed. Second, our prognosis assessment model mainly relies on RNA sequencing data from the TCGA database, which may limit the model’s general applicability. Finally, due to the lack of functional validation from clinical, cellular, and animal model aspects, the reliability of our data analysis results needs further confirmation.

This study demonstrates that the selected features are strongly associated with overall survival in LUAD patients, showing consistent prognostic value across training, test sets, and subgroups. Their correlation with clinicopathological parameters further supports their potential as reliable tools for risk stratification and personalized treatment.

## Data Availability

The original contributions presented in the study are included in the article/**Supplementary Material**, further inquiries can be directed to the corresponding author.
