# Supplementary material for: Construction and validation of immune prognosis model for lung adenocarcinoma based on machine learning
Source: Front Oncol. 2025 Jul 22;15:1630663. doi: 10.3389/fonc.2025.1630663 (PMC12321856; doi:10.3389/fonc.2025.1630663)
Supplement: Supplementary file 1 [file Table1.docx]

**1.DEG**

library(tidyverse)

library(GEOquery)

library(stringr)

exp <- read.table("exp.txt",sep = "\t",row.names = 1,check.names = F,stringsAsFactors = F,header = T)

pdata <- read.table(".txt",sep = "\t",check.names = F,stringsAsFactors = F,header = T,row.names = 1)

group_list <- ifelse(str_detect(pdata$group, "APA"), "tumor",

"normal")

group_list = factor(group_list,

levels = c("normal","tumor"))

library(limma)

design=model.matrix(~group_list)

fit=lmFit(exp,design)

fit=eBayes(fit)

deg=topTable(fit,coef=2,number = Inf)

write.table(deg, file = "deg_all.txt",sep = "\t",row.names = T,col.names = NA,quote = F)

**2.LASSO**

library(survival)

library(glmnet)

library(ggplot2)

library(ggsci)

library(patchwork)

library(limma)

inputFile=".txt"

C="C"

rt=read.table(inputFile, header=T, sep="\t", check.names=F)

rt=as.matrix(rt)

rownames(rt)=rt[,1]

exp=rt[,2:ncol(rt)]

dimnames=list(rownames(exp),colnames(exp))

data=matrix(as.numeric(as.matrix(exp)),nrow=nrow(exp),dimnames=dimnames)

data=avereps(data)

data=t(data)

data=data[,read.table("disease.txt", header=F, sep="\t", check.names=F)[,1]]

sample=read.table("sample.txt",sep="\t",header=F,check.names=F,row.names = 1)

data=data[rownames(sample),]

x=as.matrix(data)

afcon=sum(sample[,1]==C)

group=c(rep("0",afcon),rep("1",nrow(data)-afcon))

group=as.matrix(group)

rownames(group)=rownames(data)

y=as.matrix(group[,1])

set.seed(123)

cvfit = cv.glmnet(x, y,family = "binomial", nlambda=100, alpha=1,nfolds = 10)

fit <- glmnet(x,y,family = "binomial")

cvfit$lambda.min

coef <- coef(fit, s = cvfit$lambda.min)

index <- which(coef != 0)

actCoef <- coef[index]

lassoGene=row.names(coef)[index]

geneCoef=cbind(Gene=lassoGene, Coef=actCoef)

write.table(geneCoef, file="geneCoef.xls", sep="\t", quote=F, row.names=F)

write.table(file="lassoset.txt",lassoGene,sep="\t",quote=F,col.names=F,row.names=F)

pdf("lasso.pdf",height = 5,width = 7)

layout(matrix(c(1,1,2,2), 2, 2, byrow = F))

plot(fit,xvar = 'lambda')

plot(cvfit)

abline(v=log(c(cvfit$lambda.min,cvfit$lambda.1se)),lty="dashed")

dev.off()

**3.randomForest**

library(randomForest)

library(limma)

library(ggpubr)

set.seed(123)

inputFile=".txt"

C="C"

rt=read.table(inputFile, header=T, sep="\t", check.names=F)

rt=as.matrix(rt)

rownames(rt)=rt[,1]

exp=rt[,2:ncol(rt)]

dimnames=list(rownames(exp),colnames(exp))

data=matrix(as.numeric(as.matrix(exp)),nrow=nrow(exp),dimnames=dimnames)

data=avereps(data)

data=t(data)

data=data[,read.table("disease.txt", header=F, sep="\t", check.names=F)[,1]]

sample=read.table("sample.txt",sep="\t",header=F,check.names=F,row.names = 1)

data=data[rownames(sample),]

colnames(data)=gsub("-", "afaf", colnames(data))

afcon=sum(sample[,1]==C)

group=c(rep("con",afcon),rep("treat",nrow(data)-afcon))

rf=randomForest(as.factor(group)~., data=data, ntree=500)

pdf(file="forest.pdf", width=6, height=6)

plot(rf, main="Random forest", lwd=2)

dev.off()

optionTrees=which.min(rf$err.rate[,1])

optionTrees

rf2=randomForest(as.factor(group)~., data=data, ntree=optionTrees)

importance=importance(x=rf2)

importance=as.data.frame(importance)

importance$size=gsub("-", "afaf", importance$size)

importance$size=rownames(importance)

importance=importance[,c(2,1)]

names(importance)=c("Gene","importance")

af=importance[order(importance$importance,decreasing = T),]

af=af[1:20,]

p=ggdotchart(af, x = "Gene", y = "importance",

color = "importance", # Custom color palette

sorting = "descending",

add = "segments",

add.params = list(color = "lightgray", size = 2),

dot.size = 6,

font.label = list(color = "white", size = 9,

vjust = 0.5),

ggtheme = theme_bw() ,

rotate=TRUE )

p1=p+ geom_hline(yintercept = 0, linetype = 2, color = "lightgray")+

gradient_color(palette =c(ggsci::pal_npg()(2)[2],ggsci::pal_npg()(2)[1]) ) +

grids()

pdf(file="importance.pdf", width=6, height=6)

print(p1)

dev.off()

rfGenes=importance[order(importance[,"importance"], decreasing = TRUE),]

write.table(rfGenes, file="rfGenes.xls", sep="\t", quote=F, col.names=T, row.names=F)

**4.SVM-RFE**

library(tidyverse)

library(glmnet)

source('msvmRFE.R')

library(VennDiagram)

library(sigFeature)

library(e1071)

library(caret)

library(randomForest)

library(limma)

inputFile=".txt"

C="C"

rt=read.table(inputFile, header=T, sep="\t", check.names=F)

rt=as.matrix(rt)

rownames(rt)=rt[,1]

exp=rt[,2:ncol(rt)]

dimnames=list(rownames(exp),colnames(exp))

data=matrix(as.numeric(as.matrix(exp)),nrow=nrow(exp),dimnames=dimnames)

data=avereps(data)

data=t(data)

data=data[,read.table("disease.txt", header=F, sep="\t", check.names=F)[,1]]

sample=read.table("sample.txt",sep="\t",header=F,check.names=F,row.names = 1)

data=data[rownames(sample),]

afcon=sum(sample[,1]==C)

group=c(rep("0",afcon),rep("1",nrow(data)-afcon))

group=as.matrix(as.numeric(group))

rownames(group)=rownames(data)

colnames(group)="Type"

input <- as.data.frame(cbind(group,data))

input$Type=as.factor(input$Type)

svmRFE(input, k = 10, halve.above = 100)

nfold = 10

nrows = nrow(input)

folds = rep(1:nfold, len=nrows)[sample(nrows)]

folds = lapply(1:nfold, function(x) which(folds == x))

results = lapply(folds, svmRFE.wrap, input, k=10, halve.above=100)

top.features = WriteFeatures(results, input, save=F) head(top.features)

write.csv(top.features,"feature_svm.csv")

featsweep = lapply(1:X, FeatSweep.wrap, results, input)

no.info = min(prop.table(table(input[,1])))

errors = sapply(featsweep, function(x) ifelse(is.null(x), NA, x$error))

pdf("svm-error.pdf",width = 5,height = 5)

PlotErrors(errors, no.info=no.info)

dev.off()

pdf("svm-accuracy.pdf",width = 5,height = 5)

Plotaccuracy(1-errors,no.info=no.info)

dev.off()

which.min(errors)

**5.ssGSEA**

library(GSVA)

library(limma)

library(GSEABase)

expFile=".txt"

gmtFile=""

rt=read.table(expFile, header=T, sep="\t", check.names=F)

rt=as.matrix(rt)

rownames(rt)=rt[,1]

exp=rt[,2:ncol(rt)]

dimnames=list(rownames(exp),colnames(exp))

mat=matrix(as.numeric(as.matrix(exp)),nrow=nrow(exp),dimnames=dimnames)

mat=avereps(mat)

mat=normalizeBetweenArrays(mat)

mat=mat[rowMeans(mat)>0,]

geneSet=getGmt(gmtFile, geneIdType=SymbolIdentifier())

ssgseaScore=gsva(mat, geneSet, method='ssgsea', kcdf='Gaussian', abs.ranking=TRUE)

normalize=function(x){

return((x-min(x))/(max(x)-min(x)))}

ssgseaOut=normalize(ssgseaScore)

ssgseaOut=rbind(id=colnames(ssgseaOut),ssgseaOut)

write.table(ssgseaOut, file="ssGSEAscore.txt", sep="\t", quote=F, col.names=F)

**6.WGCNA**

install.packages("BiocManager")

BiocManager::install("preprocessCore")

BiocManager::install("impute")

install.packages("WGCNA")

library("tidyverse")

library("WGCNA")

exp <- read.table("LIHC_fpkm_mRNA_01A.txt", sep = "\t",row.names = 1,check.names = F,header = T)

datExpr0 = as.data.frame(t(exp))

gsg = goodSamplesGenes(datExpr0, verbose = 3)

gsg$allOK

if (!gsg$allOK){

# Optionally, print the gene and sample names that were removed:

if (sum(!gsg$goodGenes)>0)

printFlush(paste("Removing genes:", paste(names(datExpr0)[!gsg$goodGenes], collapse = ", ")));

if (sum(!gsg$goodSamples)>0)

printFlush(paste("Removing samples:", paste(rownames(datExpr0)[!gsg$goodSamples], collapse = ", ")));

# Remove the offending genes and samples from the data:

datExpr0 = datExpr0[gsg$goodSamples, gsg$goodGenes]

}

sampleTree = hclust(dist(datExpr0), method = "average")

# 画图

par(cex = 0.6)

par(mar = c(0,4,2,0))

plot(sampleTree)

plot(sampleTree, main = "Sample clustering to detect outliers", sub="", xlab="", cex.lab = 1.5, cex.axis = 1.5, cex.main = 2)

abline(h = XX, col = "red")

clust = cutreeStatic(sampleTree, cutHeight =XX, minSize = 10)

table(clust)

keepSamples = (clust==1)

datExpr0 = datExpr0[keepSamples, ]

dev.off()

sampleTree2 = hclust(dist(datExpr0), method = "average")

plot(sampleTree2)

enableWGCNAThreads()

powers = c(1:20)

sft = pickSoftThreshold(datExpr0, powerVector = powers, verbose = 5)

par(mfrow = c(1,2))

cex1 = 0.9

plot(sft$fitIndices[,1], -sign(sft$fitIndices[,3])*sft$fitIndices[,2],

xlab="Soft Threshold (power)",ylab="Scale Free Topology Model Fit,signed R^2",type="n",

main = paste("Scale independence"));

text(sft$fitIndices[,1], -sign(sft$fitIndices[,3])*sft$fitIndices[,2],

labels=powers,cex=cex1,col="red");

abline(h=XX,col="red")

plot(sft$fitIndices[,1], sft$fitIndices[,5],

xlab="Soft Threshold (power)",ylab="Mean Connectivity", type="n",

main = paste("Mean connectivity"))

text(sft$fitIndices[,1], sft$fitIndices[,5], labels=powers, cex=cex1,col="red")

softPower =sft$powerEstimate

softPower = XX

adjacency = adjacency(datExpr0, power = softPower)

TOM = TOMsimilarity(adjacency)

dissTOM = 1-TOM

save(TOM,file = "TOM.Rda")

geneTree = hclust(as.dist(dissTOM), method = "average");

plot(geneTree, xlab="", sub="", main = "Gene clustering on TOM-based dissimilarity",

labels = FALSE, hang = 0.04)

minModuleSize = 30

dynamicMods = cutreeDynamic(dendro = geneTree, distM = dissTOM,

deepSplit = 2, pamRespectsDendro = FALSE,

minClusterSize = minModuleSize);

table(dynamicMods)

dynamicColors = labels2colors(dynamicMods)

table(dynamicColors)

plotDendroAndColors(geneTree, dynamicColors, "Dynamic Tree Cut",

dendroLabels = FALSE, hang = 0.03,

addGuide = TRUE, guideHang = 0.05,

main = "Gene dendrogram and module colors")

MEList = moduleEigengenes(datExpr0, colors = dynamicColors)

MEs = MEList$eigengenes

MEDiss = 1-cor(MEs);

METree = hclust(as.dist(MEDiss), method = "average")

plot(METree, main = "Clustering of module eigengenes",

xlab = "", sub = "")

MEDissThres = 0.1

abline(h=MEDissThres, col = "red")

merge = mergeCloseModules(datExpr0, dynamicColors, cutHeight = MEDissThres, verbose = 3)

mergedColors = merge$colors

mergedMEs = merge$newMEs

plotDendroAndColors(geneTree, mergedColors,"Dynamic Tree Cut",

dendroLabels = FALSE, hang = 0.03,

addGuide = TRUE, guideHang = 0.05,

main = "Gene dendrogram and module colors")

moduleColors = mergedColors

table(moduleColors)

colorOrder = c("grey", standardColors(50))

moduleLabels = match(moduleColors, colorOrder)-1

MEs = mergedMEs

dev.off()

clinical <- read.table("XX.txt",sep = "\t",row.names = 1,check.names = F,stringsAsFactors = F,header = T)

clinical <- clinical[rownames(datExpr0),]

identical(rownames(clinical),rownames(datExpr0))

head(clinical)

datTraits = as.data.frame(do.call(cbind,lapply(clinical, as.numeric)))

rownames(datTraits) = rownames(clinical)

sampleTree2 = hclust(dist(datExpr0), method = "average")

traitColors = numbers2colors(datTraits, signed = FALSE)

plotDendroAndColors(sampleTree2,

traitColors,

groupLabels = names(datTraits),

main = "Sample dendrogram and trait heatmap")

dev.off()

MEs=orderMEs(MEs)

textMatrix=paste(signif(moduleTraitCor,2),"\n(",signif(moduleTraitPvalue,1),")",sep="")

dim(textMatrix)=dim(moduleTraitCor)

labeledHeatmap(Matrix=moduleTraitCor,

xLabels=colnames(datTraits),

yLabels=names(MEs),

ySymbols=names(MEs),

colorLabels=FALSE,

colors=blueWhiteRed(50),

textMatrix=textMatrix,

setStdMargins=FALSE,

cex.text=0.7,

cex.lab=0.7,

zlim=c(-1,1),

main=paste("Module-trait relationships"))

dev.off()

modNames = substring(names(MEs), 3)

geneModuleMembership = as.data.frame(cor(datExpr0, MEs, use = "p"))

a <- geneModuleMembership

a <- a %>% rownames_to_column()

MMPvalue = as.data.frame(corPvalueStudent(as.matrix(geneModuleMembership), nSamples))

names(geneModuleMembership) = paste("MM", modNames, sep="")

names(MMPvalue) = paste("p.MM", modNames, sep="")

traitNames=names(datTraits)

geneTraitSignificance = as.data.frame(cor(datExpr0, datTraits, use = "p"))

GSPvalue = as.data.frame(corPvalueStudent(as.matrix(geneTraitSignificance), nSamples))

names(geneTraitSignificance) = paste("GS.", traitNames, sep="")

names(GSPvalue) = paste("p.GS.", traitNames, sep="")

for (trait in traitNames){

traitColumn=match(trait,traitNames)

for (module in modNames){

column = match(module, modNames)

moduleGenes = moduleColors==module

if (nrow(geneModuleMembership[moduleGenes,]) > 1){

outPdf=paste(trait, "_", module,".pdf",sep="")

pdf(file=outPdf,width=7,height=7)

par(mfrow = c(1,1))

verboseScatterplot(abs(geneModuleMembership[moduleGenes, column]),

abs(geneTraitSignificance[moduleGenes, traitColumn]),

xlab = paste("Module Membership in", module, "module"),

ylab = paste("Gene significance for ",trait),

main = paste("Module membership vs. gene significance\n"),

cex.main = 1.2, cex.lab = 1.2, cex.axis = 1.2, col = module)

abline(v=0.8,h=0.5,col="red")

dev.off()

}

}

}

for (mod in 1:nrow(table(moduleColors)))

{

modules = names(table(moduleColors))[mod]

probes = colnames(datExpr0)

inModule = (moduleColors == modules)

modGenes = probes[inModule]

write.table(modGenes, file =paste0(modules,".txt"),sep="\t",row.names=F,col.names=F,quote=F)

}

**7.Prognostic analysis**

#install.packages('survival')

#install.packages("UpSetR")

#library(UpSetR)

pFilter=0.05

setwd("")

library(survival)

rt <- t(rt)

rt <- as.data.frame(rt)

rt <- na.omit(rt)

rt=read.table("xx.txt",header=T,sep="\t",check.names=F,row.names=1)

outTab=data.frame()

for(i in colnames(rt[,3:ncol(rt)])){

cox <- coxph(Surv(futime, fustat) ~ rt[,i], data = rt)

coxSummary = summary(cox)

coxP=coxSummary$coefficients[,"Pr(>|z|)"]

outTab=rbind(outTab,

cbind(id=i,

z=coxSummary$coefficients[,"z"],

HR=coxSummary$conf.int[,"exp(coef)"],

HR.95L=coxSummary$conf.int[,"lower .95"],

HR.95H=coxSummary$conf.int[,"upper .95"],

pvalue=coxSummary$coefficients[,"Pr(>|z|)"])

)

}

outTab = outTab[is.na(outTab$pvalue)==FALSE,]

outTab=outTab[order(as.numeric(as.vector(outTab$pvalue))),]

write.table(outTab,file="xx.txt",sep="\t",row.names=F,quote=F)#画火山图

sigTab=outTab[as.numeric(as.vector(outTab$pvalue))<pFilter,]

write.table(sigTab,file="xx.txt",sep="\t",row.names=F,quote=F)

sigGenes=c("futime","fustat")

sigGenes=c(sigGenes,as.vector(sigTab[,1]))

uniSigExp=rt[,sigGenes]

uniSigExp=cbind(id=row.names(uniSigExp),uniSigExp)

write.table(uniSigExp,file="xx.txt",sep="\t",row.names=F,quote=F)

library(ggplot2)

setwd("")

rt = read.table("xx.txt",header=T,sep="\t")

pdf(file="mRNA.vol-M.pdf",width = 5.5,height = 6)

yMax=8

xMax=5

plot(rt$z,-log10(rt$pvalue),

ylab="-log10(pvalue)",xlab="z-score",

ylim=c(0,yMax),xlim=c(-xMax,xMax),

bty="l",col="skyblue",

yaxs="i",pch=20, cex=0.8)

diffSub=subset(rt, pvalue<0.05)

points(diffSub$z,-log10(diffSub$pvalue), pch=20, col="red",cex=1)

abline(v=0,lty=2,lwd=1.5)

legend("topright",

c("Prognosis-related RNA", "No significant"),

bty="n",

pch=19,

col=c("red","skyblue"))

dev.off()

**8.GSEA**

library(ggplot2)

library(limma)

library(pheatmap)

library(ggsci)

lapply(c('clusterProfiler','enrichplot','patchwork'), function(x) {library(x, character.only = T)})

library(org.Hs.eg.db)

library(patchwork)

expFile=".txt"

hub="LASSO.txt"

rt=read.table(expFile,sep="\t",header=T,check.names=F)

rt=as.matrix(rt)

rownames(rt)=rt[,1]

exp=rt[,2:ncol(rt)]

dimnames=list(rownames(exp),colnames(exp))

data=matrix(as.numeric(as.matrix(exp)),nrow=nrow(exp),dimnames=dimnames)

data=avereps(data)

geneaf=read.table(hub,sep="\t",header=F,check.names=F)[,1]

for (genei in geneaf) {

group <- ifelse(data[c(genei),]> median(data[c(genei),]), "High", "Low")

group <- factor(group,levels = c("High","Low"))

design <- model.matrix(~0+group)

colnames(design) <- levels(group)

fit <- lmFit(data,design)

cont.matrix<-makeContrasts(High-Low,levels=design)

fit2 <- contrasts.fit(fit, cont.matrix)

fit2 <- eBayes(fit2)

deg=topTable(fit2,adjust='fdr',number=nrow(data))

Diff=deg

Diff=Diff[order(as.numeric(as.vector(Diff$logFC))),]

diffGene=as.vector(rownames(Diff))

diffLength=length(diffGene)

afGene=c()

if(diffLength>(60)){

afGene=diffGene[c(1:30,(diffLength-30+1):diffLength)]

}else{

afGene=diffGene

}

afExp=data[afGene,]

Type1=as.data.frame(group)

Type1=Type1[order(Type1$group,decreasing = T),,drop=F]

Type=Type1[,1]

names(Type)=rownames(Type1)

Type=as.data.frame(Type)

anncolor=list(Type=c(High="red",Low="blue" ))

logFC_t=0

deg$g=ifelse(deg$P.Value>0.05,'stable',

ifelse( deg$logFC > logFC_t,'UP',

ifelse( deg$logFC < -logFC_t,'DOWN','stable') )

)

table(deg$g)

deg$symbol=rownames(deg)

df <- bitr(unique(deg$symbol), fromType = "SYMBOL",

toType = c( "ENTREZID"),

OrgDb = org.Hs.eg.db)

DEG=deg

DEG=merge(DEG,df,by.y='SYMBOL',by.x='symbol')

data_all_sort <- DEG %>%

arrange(desc(logFC))

geneList = data_all_sort$logFC

names(geneList) <- data_all_sort$ENTREZID

head(geneList)

kk2 <- gseKEGG(geneList = geneList,

organism = 'hsa',

nPerm = 10000,

minGSSize = 10,

maxGSSize = 200,

pvalueCutoff = 0.05,

pAdjustMethod = "none" )

class(kk2)

colnames(kk2@result)

kegg_result <- as.data.frame(kk2)

rownames(kk2@result)[head(order(kk2@result$enrichmentScore))]

af=as.data.frame(kk2@result)

write.table(af,file=paste0("2.",paste0(genei,"_all_GSEA.xls")),sep="\t",quote=F,col.names=T)

num=5

pdf(paste0("2.",paste0(genei,"_down_GSEA.pdf")),width = 8,height = 8)

af=gseaplot2(kk2, geneSetID = rownames(kk2@result)[head(order(kk2@result$enrichmentScore),num)])

print(af)

dev.off()

pdf(paste0("2.",paste0(genei,"_up_GSEA.pdf")),width = 8,height = 8)

af=gseaplot2(kk2, geneSetID = rownames(kk2@result)[tail(order(kk2@result$enrichmentScore),num)])

print(af)

dev.off()

num=5

pdf(paste0("2.",paste0(genei,"_all_GSEA.pdf")),width = 4,height = 5)

af=gseaplot2(kk2, geneSetID = rownames(kk2@result)[c(head(order(kk2@result$enrichmentScore),num),tail(order(kk2@result$enrichmentScore),num))])

print(af)

dev.off()

}

**9.ANN**

zscale=read.table()

ind<-sample(2,nrow(zscale),replace = T,prob = c()

trainset<-zscale[ind==1,]

testset<-zscale[ind==2,]

trainset$normal<-trainset$group=="normal"

trainset$tumor<-trainset$group=="tumor"

trainset1 = trainset[,2:6]

testset1 = testset[,2:4]

library(neuralnet)

library(NeuralNetTools)

network<-neuralnet(normal + tumor ~ X + X +X ,trainset1,hidden = c())

par(cex = 0.8)

plotnet(network,pos_col = "red", neg_col = "grey")

par(mfrow=c(2,3))

gwplot(network,selected.covariate = "X")

gwplot(network,selected.covariate = "X")

gwplot(network,selected.covariate = "X")

net.predict1<-compute(network,trainset1[,2:4])$net.result

net.prediction1<-c("normal","tumor")[apply(net.predict1,1,which.max)]

predict.table1<-table(trainset$group,net.prediction1)

net.predict<-compute(network,testset[,2:4])$net.result

net.prediction<-c("normal","tumor")[apply(net.predict,1,which.max)]

predict.table<-table(testset$group,net.prediction)

write.table (trainset,file ="trainset.txt", row.names = T, col.names =T, quote =FALSE)

write.table (testset,file ="testset.txt", row.names = T, col.names =T, quote =FALSE)

cbind(net.prediction1,trainset[,2])

cbind(net.prediction,testset[,2])

predict.table1

net.prediction1

net.predict1

confusionMatrix(predict.table1)

predict.table

net.prediction

net.predict
