# Supplementary material for: Construction and validation of immune prognosis model for lung adenocarcinoma based on machine learning
Source: Front Oncol. 2025 Jul 22;15:1630663. doi: 10.3389/fonc.2025.1630663 (PMC12321856; doi:10.3389/fonc.2025.1630663)
Supplement: Supplementary file 2 [file Table2.docx]

| **Indicators** | **TCGA Training Set**  **55 T + 38N** | **TCGA Validation Set**  **30 T + 30 N** | **External validation set**  **10 T + 10 N** |
| --- | --- | --- | --- |
| Accuracy | 0.946 | 0.850 | 0.900 |
| Precision | 0.963 | 0.839 | 0.900 |
| Recall | 0.945 | 0.867 | 0.900 |
| F1-score | 0.954 | 0.853 | 0.900 |
| AUC | 0.929 | 0.876 | 0.906 |

**Five indicators to evaluate ANN**
